# ß-Adrenergic Stimulation Increases RyR2 Activity via Intracellular Ca^2+^ and Mg^2+^ Regulation

**DOI:** 10.1371/journal.pone.0058334

**Published:** 2013-03-22

**Authors:** Jiao Li, Mohammad S. Imtiaz, Nicole A. Beard, Angela F. Dulhunty, Rick Thorne, Dirk F. vanHelden, Derek R. Laver

**Affiliations:** 1 School of Biomedical Sciences and Pharmacy, University of Newcastle and Hunter Medical Research Institute, Callaghan, New South Wales, Australia; 2 John Curtin School of Medical Research, Australian National University, Canberra, Australian Capital Territory, Australia; University of Queensland, Australia

## Abstract

Here we investigate how ß**-**adrenergic stimulation of the heart alters regulation of ryanodine receptors (RyRs) by intracellular Ca^2+^ and Mg^2+^ and the role of these changes in SR Ca^2+^ release. RyRs were isolated from rat hearts, perfused in a Langendorff apparatus for 5 min and subject to 1 min perfusion with 1 µM isoproterenol or without (control) and snap frozen in liquid N_2_ to capture their phosphorylation state. Western Blots show that RyR2 phosphorylation was increased by isoproterenol, confirming that RyR2 were subject to normal ß**-**adrenergic signaling. Under basal conditions, S2808 and S2814 had phosphorylation levels of 69% and 15%, respectively. These levels were increased to 83% and 60%, respectively, after 60 s of ß**-**adrenergic stimulation consistent with other reports that ß**-**adrenergic stimulation of the heart can phosphorylate RyRs at specific residues including S2808 and S2814 causing an increase in RyR activity. At cytoplasmic [Ca^2+^] <1 µM, ß**-**adrenergic stimulation increased luminal Ca^2+^ activation of single RyR channels, decreased luminal Mg^2+^ inhibition and decreased inhibition of RyRs by mM cytoplasmic Mg^2+^. At cytoplasmic [Ca^2+^] >1 µM, ß**-**adrenergic stimulation only decreased cytoplasmic Mg^2+^ and Ca^2+^ inhibition of RyRs. The *K_a_* and maximum levels of cytoplasmic Ca^2+^ activation site were not affected by ß**-**adrenergic stimulation.

Our RyR2 gating model was fitted to the single channel data. It predicted that in diastole, ß**-**adrenergic stimulation is mediated by 1) increasing the activating potency of Ca^2+^ binding to the luminal Ca^2+^ site and decreasing its affinity for luminal Mg^2+^ and 2) decreasing affinity of the low-affinity Ca^2+^/Mg^2+^ cytoplasmic inhibition site. However in systole, ß**-**adrenergic stimulation is mediated mainly by the latter.

## Introduction

Exercise and stress induce the sympathetic “fight or flight” response that increases heart rate and contractility. During this response, increased catecholamine concentrations stimulate cardiac β-adrenergic receptors, resulting in adenylyl cyclase activation, increased cyclic AMP and increased activity of cyclic AMP-dependent protein kinase A (PKA). Increased intracellular [Ca^2+^] causes Ca^2+^/calmodulin dependent protein kinase II (CaMKII) autophosphorylation so that it remains active at low [Ca^2+^] [1], [2]. PKA and CaMKII phosphorylation increase Ca^2+^-transport activity that underlies contraction and pacemaking in cardiac cells [1], [3]–[6].

Systolic contraction follows activation of sarcolemmal voltage-gated L-type Ca^2+^ channels during an action potential resulting in Ca^2+^-influx, which activates cardiac ryanodine receptor (RyR2) calcium release channels on the sarcoplasmic reticulum (SR, major intracellular Ca^2+^ store). The subsequent rise in cytoplasmic [Ca^2+^] causes contraction. Diastolic relaxation occurs with cessation of Ca^2+^ release and Ca^2+^ sequestration by the SR Ca^2+^ uptake transporter (sarcoplasmic/endoplasmic reticulum Ca^2+^-ATPase) [7].

Three phosphorylation sites have been confirmed by ^32^P incorporation assays and known to be phosphorylated *in vivo*; namely S2808/S2809, S2814/S2815 (depending on the species) and S2030 [2], [8], [9]. Mass spectroscopy of the RyR2 phosphorylation domain (aa2699–2904) has revealed additional, *in vitro* phosphorylation sites on RyR2 [10], [11] at S2810, S2811, S2797 and T2876. Present understanding of the contribution of RyR2 to ß**-**adrenergic stimulation is limited by a lack of knowledge of how RyR2 is regulated by phosphorylation within the cell. Most investigations show increased RyR2 activity in lipid bilayers with *in vitro* phosphorylation at these sites by exogenous PKA and CaMKII [8], [12] (although [13] reports that CaMKII inhibits RyR2), increasing RyR2 sensitivity to luminal Ca^2+^ [14] and decreasing cytoplasmic Mg^2+^ inhibition [12]. However, it is not clear if exogenous enzymes can replicate the mechanisms of RyR2 phosphorylation at rest or during ß**-**adrenergic stimulation. Therefore, just how ß-adrenergic signaling alters regulation of RyR2 by luminal and cytoplasmic Ca^2+^ and Mg^2+^ is unknown. In this study, we perfused isolated rat hearts with the ß-adrenergic agonist, isoproterenol, then incorporated RyR2 from these hearts into artificial lipid bilayers whilst preserving their phosphorylation state. The response of RyRs to luminal and cytoplasmic Ca^2+^ and Mg^2+^ indicated significant and novel changes in RyR2 function associated with increased RyR2 phosphorylation at S2808 and S2814 induced by ß**-**adrenergic stimulation. Since it is not feasible to measure RyR2 regulation under diastolic [Ca^2+^] and [Mg^2+^] (their activity is too low), we used a RyR2 gating model to predict the effects of ß**-**adrenergic stimulation on RyR2 activity within cells under diastolic conditions.

## Methods

### Perfusion of Isolated Rat Hearts

Healthy male adult rats (Sprague-Dawley) were heparinized (2000 U, injection BP), and anesthetized with Isoflurane. Hearts were rapidly removed and immediately perfused via the Langendorff method (see Methods S1 in File S1). Hearts were perfused with Krebs Henseleit buffer for 5 min after which they were either perfused in buffer with or without 1 µM isoproterenol for 1 min, while monitoring heart performance. Following perfusion, hearts were rapidly frozen in liquid nitrogen to preserve phosphorylation and stored at −80°C.

### SR Vesicle Preparation

Liquid nitrogen-frozen hearts were homogenized in buffer containing 10 mM imidazole, 0.5 mM DTT, 3 mM sodium azide, 0.29 M sucrose, 1 µg/ml leupeptin, 1 µg/ml pepstatin A, 1 mM benzamidine, 0.5 mM PMSF, 20 mM NaF, pH 6.9 (homogenization buffer). Homogenization was carried in 3×15 s bursts with a rotor-stator homogenizer (HD Scientific) followed by 10 manual strokes of a loose glass/glass Dounce homogenizer. The homogenate was then centrifuged at 7000 rpm for 20 min in Beckman Optima L-100XP Ultracentrifuge. The resulting supernatant was centrifuged at 50000 rpm for 30 min. The pellet from this step was re-homogenized with a tight manual glass/glass Dounce homogenizer in homogenization buffer also containing 0.65 M KCl (storage buffer, pH 6.7), incubated for 30 min and then centrifuged at 7000 rpm for 15 min. The supernatant was centrifuged at 50000 rpm for 1 h, and the resulting pellet was resuspended in storage buffer, snap frozen in liquid nitrogen and stored at −80°C. The NaF present in our buffers was to prevent ongoing activity of phosphatases (PP1 and PP2A) during SR vesicle preparation. The whole procedure was carried out at 4°C.

### Single Channel Recordings

Lipid bilayers were formed form a mixture of phosphatidylethanolamine and phosphatidylcholine (8∶2 wt/wt) in 50 mg/ml n-decane. Lipid bilayers were formed across a delrin hole (approximately 100 µm diameter), separating two experimental compartments, *cis* (cytoplasmic) and *trans* (luminal). For vesicle fusions the *cis* solution contained (mM): 250 Cs^+^ (230 CsCH_3_O_3_S, 20 CsCl, both from Sigma Aldrich), 1–5 CaCl_2_ (BDH Chemicals), and 500 Mannitol (Ajax Chemicals); while *trans* solution contained (mM): 50 Cs^+^ (30 CsCH_3_O_3_S, 20 CsCl), 0.1 CaCl_2_ and 10 TES. For channel recordings, [Cs^+^] in *trans* solution was raised to 250 mM by adding an appropriate volume of 4 M CsCH_3_O_3_S. During experiments the composition of the *cis* solution was altered by local perfusion, which provided solution exchange within ∼1 s [15], and the composition of the *trans* solutions was altered by addition of aliquots of stock solutions. All experiments were performed at room temperature (21–26°C).

All solutions were made with MilliQ water and were pH buffered with 10 mM TES (*N*-tris [hydroxymethyl] methyl-2-aminoethanesulfonic acid; ICN Biomedicals) and adjusted to pH 7.4 by CsOH (ICN Biomedicals), using a TPS digital pH meter. Free [Ca^2+^] ≤3 µM was buffered using 4.5 mM BAPTA (1,2-bis (2-aminophenoxy) ethane- *N*, *N*, *N*’, *N*’- tetraacetic acid; Invitrogen) and additional 1 mM dibromo BAPTA was used for 10 µM free Ca^2+^. Sodium citrate was used to adjust free [Ca^2+^] between 10–50 µM. A Ca^2+^ electrode (Radiometer) was used to determine free [Ca^2+^] in the experimental solutions and the purity of Ca^2+^ buffers and Ca^2+^ stock solutions using titrations against a 100 mM CaCl_2_ standard (Fluka).

All recording solutions contained ATP (2 mM, sodium salt from Sigma Chemicals), which buffers Mg^2+^. The Ca^2+^ electrode was used to determine the purity of the ATP stock solutions by titrations against the 100 mM CaCl_2_ standard. Free [Mg^2+^] was estimated using published association constants [16] and the program “Bound and Determined” [17]. The free [Mg^2+^] was confirmed using the fluorescent magnesium indicator, Mag-fura*-*2 (tetra potassium salt from Molecular Probes). The ratio of fluorescence intensities at 340 and 380 nm were calibrated in the experimental solutions (250 mM Cs^+^ solutions, see above) also containing 5 µM Mag-fura*-*2, 4.5 mM BAPTA, (free [Ca^2+^] <1 µM) and MgCl_2_ from aliquots of a calibrated stock.

### SDS PAGE and Western Blotting

Standard techniques were used for SDS PAGE and Western Blotting (see Methods S1 in File S1 details). Phospho-antibodies were obtained from Badrilla (UK). To establish loading controls, each membrane probed with phospho-antibodies was re-probed with anti-RyR2 (C3–33 from Abcam) after quenching of HRP activity with sodium azide (3% w/v for 3 h at room temperature; see Figure S1 in File S2) [18]. The ratio of binding of phospho- and RyR2- antibodies was used to measure the degree of RyR2 phosphorylation.

### *In-vitro* Phosphorylation and Dephosphorylation Assays

Phosphorylation of RyR2 was determined by probing heart SR isolates using phospho-specific antibodies against S2808 and S2814 (pS2808 and pS2814, respectively). We also used an antibody against dephosphorylated S2808 (DepS2808) as a cross check for pS2808. The average degree of S2808 phosphorylation was quantified as both pS2808 immunostaining relative to maximum PKA phosphorylation and also DepS2808 staining (1- DepS2808 staining) relative to minimal phosphorylation in PP1 incubated samples. Similarly, S2814 phosphorylation was expressed as pS2814 immunostaining relative to maximal phosphorylation levels achieved using calcium calmodulin (CaCaM) to activate endogenous CaMKII. Maximal and minimal phosphorylation levels were determined from the time courses of phosphorylation during incubations with PP1, PKA and CaCaM (see Methods S1 in File S1 and Figures S2, S3, and S4 in File S2, respectively).

We tested that our anti-pS2808 and anti-pS2814 antibodies had the same specificity in detecting PKA and CaMKII phosphorylation of RyR2 as seen in a previous study [19]. We subjected PP1-dephosphorylated RyR2 to successive incubations of PKA and CamKII (or in reverse order, see Methods S1 in File S1) and followed the time course of staining by both antibodies (Figures S3 and S4 in File S2). We found that 10 min incubations with PP1 caused maximal decrease in RyR2 phosphorylation at S2808 (Figures S2, S3, and S4 in File S2). Subsequent PKA incubation for 20 min produced near maximal S2808 phosphorylation and also increased antibody staining by S2814 to 20% of maximal levels attainable with CaMKII phosphorylation. Alternatively, CamKII incubation of PP1 dephosphorylated RyRs for 10 min produced maximal S2814 phosphorylation and also increased antibody staining by S2808 to 50% of maximal levels attainable with PKA phosphorylation. Thus, antibodies for S2808 respond to CamKII mediated phosphorylation and those for S2814 respond to PKA phosphorylation as reported previously [19].

Since fusion of vesicles with lipid bilayers was performed in the absence of phosphatase inhibitors, we determined if RyRs could maintain their phosphorylation levels at S2808 during vesicle fusion by incubating vesicles in the buffers used for vesicle fusion and channel recording. We found no significant decrease in phosphorylation of S2808 during incubation with vesicle fusion buffers over a period of 30 min (Figure S5 in File S2). Since single channel experiments were performed with ATP, we determined whether ATP could phosphorylate S2814 via endogenous CaMKII/calmodulin [20] SR vesicles from control hearts incubated for 10 min in buffers containing 2 mM ATP, without exogenous CaMKII or calmodulin, did not exhibit increased S2814 phosphorylation (Figure S6 in File S2).

### Statistics

All data are shown as mean ± standard error of the mean (SEM). Significance was calculated by Student’s *t* test. p<0.05 was considered significant.

### Ethics Statement

All experimental procedures were approved by the University of Newcastle Animal Care and Ethics Committee (A-2009-153).

## Results

### Confirmation of ß-adrenergic Stimulation by Heart Rate and RyR2 Phosphorylation

Mean heart rate was 220±18 bpm (Table S1, hearts 1–14 in File S1) after 5 min perfusion with Krebs-Henseleit buffer. In six experiments, where isoproterenol (1 µM) was added to the perfusion buffer, heart rate increased by 77±9% (n = 6) to 348±23 bpm after 1 min (Table S1, hearts 9–14 in File S1).

**Figure 1:**
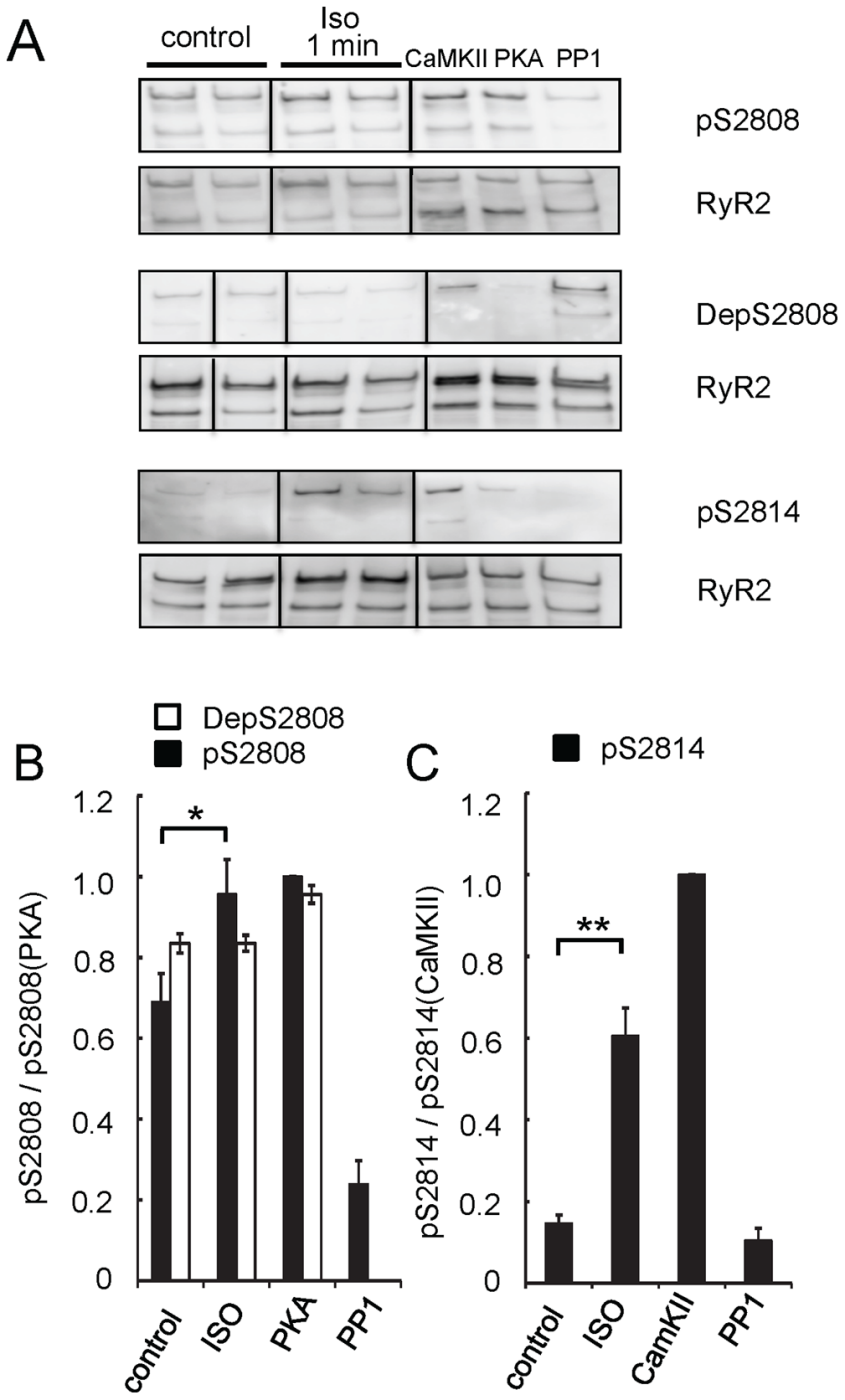
ß-adrenergic stimulation of heart phosphorylates RyR2 at S2808 and S2814. Rat hearts were perfused with buffer alone (control; examples from 2 hearts) or buffer containing 1 µM isoproterenol for 1 min (Iso 1 min; examples from 2 hearts). SR preparations, separated on tris-acetate 3–8% gel, were probed for S2808 and S2814 phosphorylation. SR isolates from control hearts were phosphorylated *in vitro* by endogenous CaMKII, exogenous PKA or dephosphorylated by exogenous PP1 (Methods). Lanes within each grouping were obtained from the same Western membranes and displayed using identical image intensity and contrast. (**A**) Representative Western blots probed with pS2808, DepS2808 or pS2814 antibodies and re-probed with anti-RyR2. (**B**) Relative levels of S2808 phosphorylation and (**C**) S2814 phosphorylation. Band densities were normalized to RyR2 loading in each lane relative to maximal band densities of PP1 for DepS2808; PKA for pS2808 and CaMKII for pS2814. Data show mean ± sem of 3–6 hearts where number of replicates for each heart are given in Table S1 in File S1 (* p<0.05, ** p<0.01).

Western blot analysis using anti-RyR2 and phospho antibodies (Figure 1A) revealed a band corresponding with RyR2 at 560 kDa (full length) and a fainter band corresponding to a 400 kDa, c-terminal fragment similar to that seen in many previous studies (e.g. [21], [22]). We measured the effects of isoproterenol perfusion on RyR2 phosphorylation to confirm that RyR2 had been subjected to normal ß-adrenergic signalling processes in the heart. ß-adrenergic stimulation of rat heart was correlated with an increased phosphorylation at S2814 and S2808. The relative levels of phosphorylation at S2814 after exposure to isoproterenol for 1 min increased 4-fold from that detected in control (p = 10^−5^). S2814 phosphorylation in control hearts was not different to RyR2 dephosphorylated by PP1 (Figure 1C, p = 0.16). RyRs from control hearts showed a relatively high level of phosphorylation at S2808. Western blots probed with pS2808 and DepS2808 indicated S2808 phosphorylation at 0.69±0.07 and 0.83±0.07 (respectively) of maximal PKA phosphorylation (Figure 1B). Isoproterenol stimulation for 1 min increased S2808 phosphorylation to 0.86±0.07 (p = 0.04) as determined by pS2808 staining. However, we could not detect increased phosphorylation using DepS2808. (Since our antibodies to S2030 showed no detectable binding to RyR2 from rat heart, either control or isoproterenol stimulated, S2030 was not examined.) The response of RyR2 phosphorylation to isoproterenol is typical of many previous studies of ß-adrenergic stimulation (see discussion).

### Effect of ß-adrenergic Stimulation on RyR2 Activity

**Figure 2:**
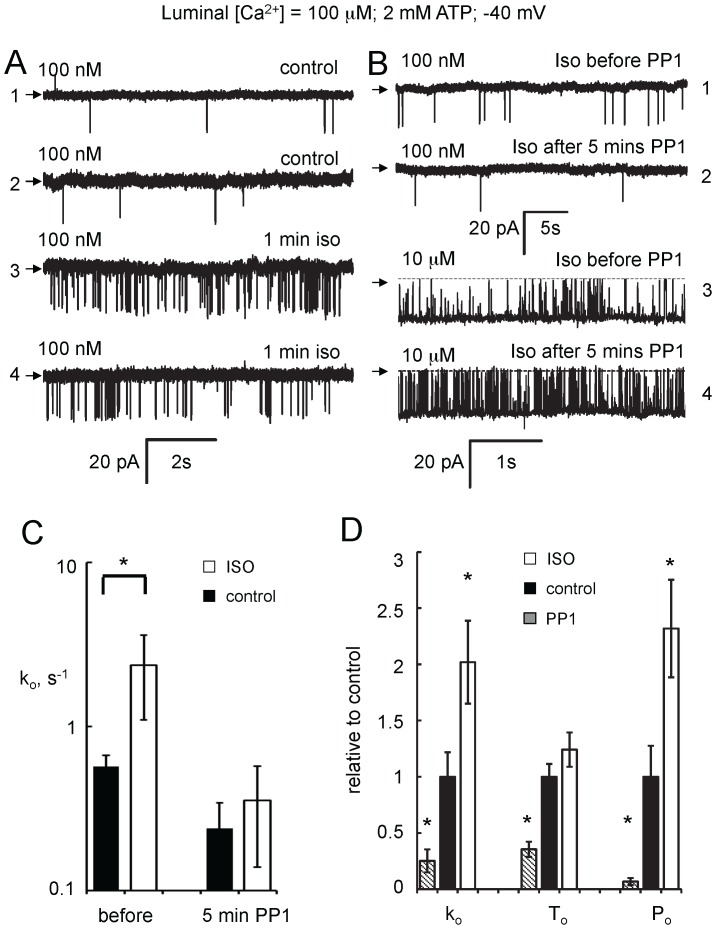
Effect of ß-adrenergic stimulation and RyR2 phosphorylation on RyR2 gating. (**A**) Representative single channel recordings of RyR2 in the presence of cytoplasmic 2 mM ATP and with cytoplasmic [Ca^2+^] at the left of each trace. RyRs were from rat hearts perfused with control buffer (Methods; Traces 1 & 2) or buffer containing 1 µM isoproterenol for 1 min (Traces 3 & 4). Each trace shows a separate experiment. At −40 mV current is downward from the baseline (arrows). (**B**) Recordings of a RyR2 channel from a 1 min isoproterenol-exposed heart, before and after incubation with cytoplasmic PP1 (7–10 units). Cytoplasmic [Ca^2+^] was 100 nM (Traces 1 & 2) and 10 µM (Traces 3 & 4). (**C**) Effect of PP1 on RyR2 opening rate at 100 nM Ca^2+^ compiled from 8 experiments like that shown in B (Traces 1 & 2). Four RyRs were used from the control and ISO groups. (**D**) Opening rate, *k_o_*, mean open duration, *T_o_* and open probability, *P_o_*, of RyR2 at 100 nM Ca^2+^, for control RyR2 treated with PP1, RyR2 from control and isoproterenol treated hearts. Mean values of *k_o_, T_o_* and *P_o_* are normalised to their respective means in RyRs from control hearts. Asterisks indicate significant differences from control (p<0.05).

We investigated the concentration dependencies of cytoplasmic and luminal Ca^2+^ and Mg^2+^ regulation of RyR2 at a bilayer potential of −40 mV where the four identified Ca^2+^/Mg^2+^ regulation mechanisms in RyR2 are best revealed [23], [24]. In initial experiments, the cytoplasmic bath contained 100 nM Ca^2+^ (diastolic [Ca^2+^]) with 2 mM ATP and the luminal bath contained 0.1 mM Ca^2+^. Figure 2A (Traces 1 and 2) shows representative single channel recordings from two experiments on RyRs from control hearts performed under these conditions. RyR2 from isoproterenol stimulated hearts showed similar gating kinetics, albeit with higher opening rates (Figure 2A, Traces 3 and 4).

RyR2 was dephosphorylated to assess whether changes in RyR2 activity after ß-adrenergic stimulation were due to increased RyR2 phosphorylation. The activity of RyR2 from control and isoproterenol stimulated hearts was measured with 100 nM and 10 µM cytoplasmic [Ca^2+^]. RyR2 was dephosphorylated in-bilayer by adding PP1 (7–10 units) to the *cis* bath for 5 min either by local perfusion or by aliquot addition of PP1 [25]. Then the *cis* chamber was perfused with solutions lacking PP1 and with cytoplasmic [Ca^2+^] of 100 nM or 10 µM and the same channel was recorded again (recordings shown in Figure 2B). In 8 of 8 experiments, PP1 reduced RyR2 opening rate at 100 nM cytoplasmic Ca^2+^ (Figure 2C). Prior to PP1 incubation, RyRs from isoproterenol treated hearts (n = 4) had significantly higher opening rates than RyRs from control hearts (n = 4). However, after PP1 incubation, there was no significant difference in opening rate between the two groups. These results suggest that ß-adrenergic stimulation increases RyR2 opening rate due to an increased channel phosphorylation. PP1 inhibition of RyR2 from control hearts suggests a significant phosphorylation-mediated RyR2 stimulation in the absence of excess ß-adrenergic stimulation. At 10 µM Ca^2+^ (n = 7), PP1 did not change either opening rate or mean open duration (p = 0.86 or 0.13, respectively). Figure 2D shows the relative differences in RyR gating parameters of three groups of RyRs; namely, 1) control RyRs that were incubated with PP1 for 5 min, 2) RyRs from control hearts and 3) RyRs from isoproterenol stimulated hearts. (Absolute values, numbers of experiments and p-values are given in Table 1). ß-Adrenergic stimulation of RyRs in control heart led to a 2.5-fold increase in open probability (*P_o_*) due to an increase in RyR opening rate whereas PP1 incubation led to a 15-fold decrease in *P_o_* due to decreases in both opening rate and mean open duration.

**Table 1:** Comparison of opening rates (1/mean closed duration), mean open durations and open probability for RyR2 from various treatment groups in Figure 2D: RyRs from control hearts incubated with PP1 for 5 min, control and stimulated with isoproterenol for 1 min.

treatment	opening rate s^−1^	mean open duration ms	open probability ×10^−3^	n
PP1	0.52±0.21 0.02*	2.8±0.5 0.04*	0.9±0.4 0.0001*	11
Control	2.0±0.5	7.9±0.9	13±4	39
ISO 1 min	4.1±0.8 0.007*	9.8±1.2 0.3	32±6 0.004*	95

Data show mean ± sem and p values for deviation from control hearts. Asterisks indicate significant difference (p<0.05). n indicates the number of experiments.

### Effect of ß-adrenergic Stimulation on Regulation of RyR2 by Ca^2+^

#### Cytoplasmic Ca^2+^ regulation

**Figure 3:**
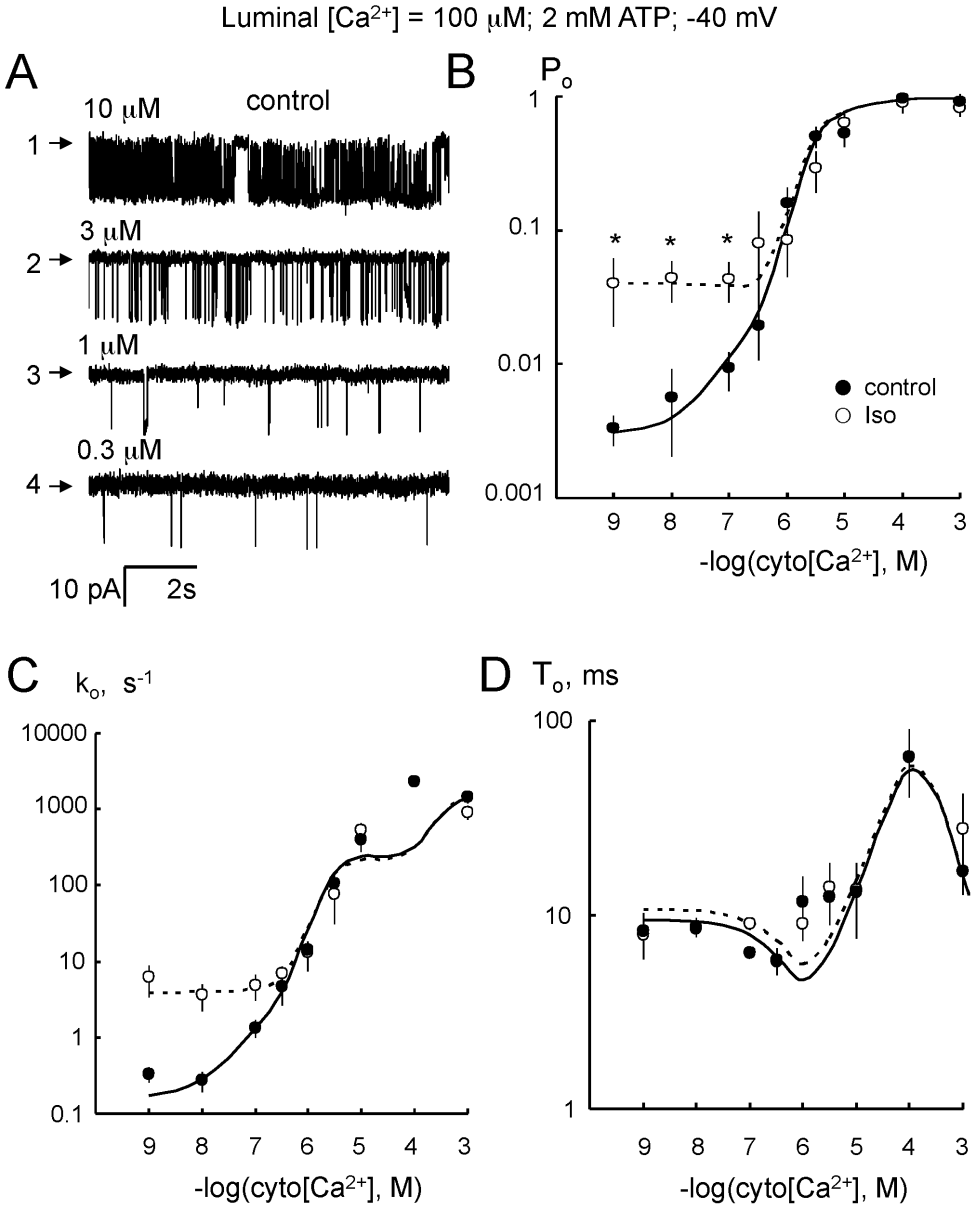
Activation of RyR2 by cytoplasmic Ca^2+^. (**A**) Single channel recordings of RyR2 from control rat hearts in the presence of cytoplasmic 2 mM ATP and with cytoplasmic [Ca^2+^] at the left of each trace. Channel openings are downward from the baseline (arrows). (**B**) Cytoplasmic Ca^2+^-dependence of open probability (*P_o_*) of RyR2 from control (•, n = 4–16) and stimulated (isoproterenol for 1 min; ○, n = 5–20) hearts. Asterisks indicate p<0.05 for differences between ○ and •. Solid and dashed curves show least-squares Hill fits to the control and isoproterenol data. (**C**) Cytoplasmic Ca^2+^-dependency of RyR2 opening rate (*k_o_*) from control (•, n = 4–16) and isoproterenol-stimulated (○ n = 4–20) hearts. (**D**) Same as C but now plotting RyR2 mean open duration (*T_o_*). Solid and dashed curves show RyR2 gating model fits to control and isoproterenol data.

RyR2 from control and isoproterenol stimulated hearts were strongly activated by micromolar cytoplasmic Ca^2+^. Increasing cytoplasmic Ca^2+^ from 0.3 µM to 10 µM decreased RyR2 mean closed duration and increased mean open duration (Figure 3A, Traces 1–4). *P_o_* exhibited activation at µM Ca^2+^ (Figure 3B) and inhibition at mM Ca^2+^ (below). Hill equations were fitted to the data (not shown), using the Hill parameters in Table 2. Isoproterenol stimulation for 1 min increased *P_o_* by 10-fold at low [Ca^2+^] (*P_min_*) with no significant effect on *K_a_* for activation or maximal Ca^2+^ activation (*P_max_*), suggesting that the Ca^2+^ sensitivity of the cytoplasmic Ca^2+^ activation site of RyR2 was not affected by isoproterenol. The Ca^2+^ dependencies of *P_o_* were also reflected in RyR2 opening rate (Figure 3C). At sub µM Ca^2+^, isoproterenol stimulation caused a 10-fold increase in opening rate and this difference became less as cytoplasmic Ca^2+^ increased. In these experiments, there was no difference in the mean open duration between the isoproterenol and control groups (p = 0.218, Figure 3D) however in the luminal Ca^2+^ experiments, differences were observed (see below).

**Table 2:** Parameter values for the Hill equation from least-squares fits to cytoplasmic and luminal Ca^2+^ regulation of *P_o_* in Figures 3, 4 and 7A.

treatment	*P_max_*	*P_min_*×10^−3^	*K_a_,* µM	*H_a_*	*n*	*K_i_,* mM	*H_i_*	*n*
cytoplasmic Ca^2+^								
Control	0.8±0.12	3.5±0.6	2.2±0.3	2±1	15	1.5±0.1	3±0.4	6
ISO (1 min)	0.8±0.11	38±4*	3.3±1.2	2±1	20	2.5±0.1*	3±0.4	5
luminal Ca^2+^								
Control	0.013±0.003	–	10±7	2†	16	0.76±0.5	1†	16
ISO (1 min)	0.064±0.01*	–	27±15	1†	33	0.51±0.2	1†	3

The Hill equation for inhibition by Ca^2+^ is: 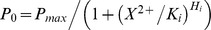. The corresponding equation for activation is: 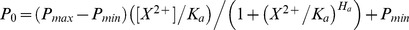
_._

* indicates significant difference from control value, p<0.05.

† indicates where *H* was not adjusted during fitting. n indicates the number of experiments.

#### Luminal Ca^2+^ regulation

**Figure 4:**
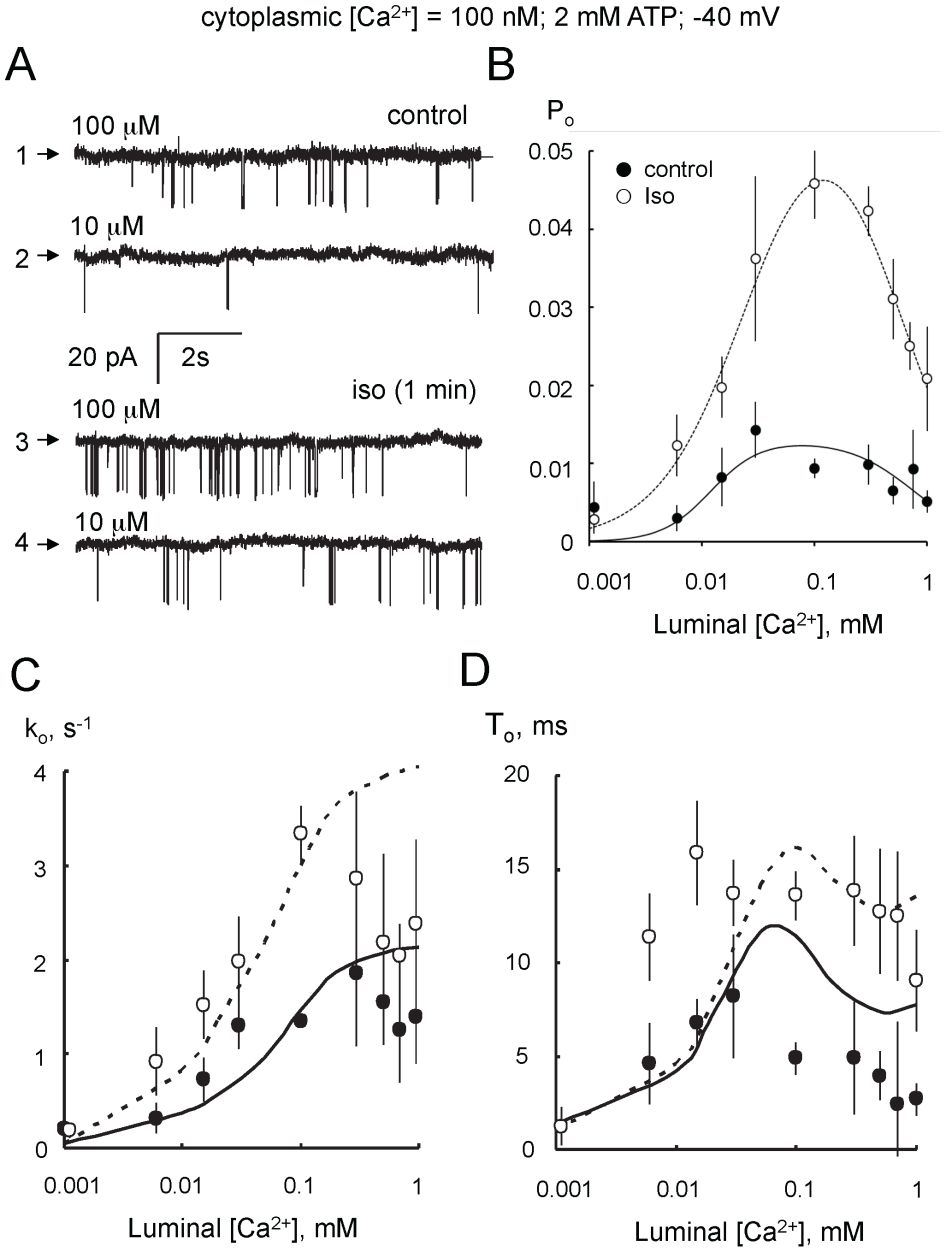
Activation of RyR2 by luminal Ca^2+^. (**A**) Single channel recordings of RyR2 from control (Traces 1 & 2) and isoproterenol stimulated hearts (1 min, Traces 3 & 4) in the presence of cytoplasmic 2 mM ATP and with luminal [Ca^2+^] at the left of each trace. (**B**) Luminal Ca^2+^-dependencies of open probability (*P_o_*) of RyR2 from control (•, n = 4–16) and isoproterenol-stimulated (○, n = 4–33) hearts. (**C**) Luminal Ca^2+^-dependencies of RyR2 opening rate (*k_o_*) from control (• n = 4–16) and isoproterenol-stimulated (○, n = 4–16) hearts. (**D**) Same as C but now plotting RyR2 mean open duration (*T_o_*)**.** Solid and dashed curves in B show least-squares Hill fits to the control and isoproterenol data. Solid and dashed curves in C & D show fits of the RyR2 gating model to data.

Figure 4A shows RyR2 activation by increasing luminal Ca^2+^ from 10 to 100 µM. Figures 4B–D show the luminal [Ca^2+^]-dependencies of the *P_o_*, opening rate (*k_o_*) and mean open time (*T_o_*) of control and isoproterenol treated RyR2 with diastolic (100 nM) cytoplasmic [Ca^2+^]. In the absence of luminal Ca^2+^, RyR2 from control and stimulated groups had a mean *P_o = _*0.003 and exhibited a bell-shaped luminal [Ca^2+^]-dependence with peak activity occurring in the presence of ∼100 µM. Peak *P_o_* of RyRs from the isoproterenol-stimulated hearts was 4-fold larger (*P_o_* = 0.045) than control (Figure 4B, curves show fits of Hill equations using Hill parameters in Table 2). The only Hill parameter that showed a significant difference between RyRs from control and stimulated hearts was *P_max_*. Therefore, isoproterenol-stimulation enhances the sensitivity of RyR2 to changes in luminal Ca^2+^ concentration without altering the *K_a_* for activation or the *K_i_* for inhibition. This enhanced sensitivity was also reflected by increases in both RyR2 opening rate and mean open duration (Figures 4C & D, curves show fits of RyR2 gating model using parameters in Table S3 in in File S1). Since measurements of opening rate are carried out on closed channels (opening rate = 1/mean closed duration), the increase in the luminal Ca^2+^-dependence of opening rate unambiguously indicates an increased effect of Ca^2+^ binding to the luminal Ca^2+^ activation site of RyR2 [26], [27].

### Effect of ß-adrenergic Stimulation on Mg^2+^ inhibition via the Luminal and Cytoplasmic Ca^2+^ Activation Sites

**Figure 5:**
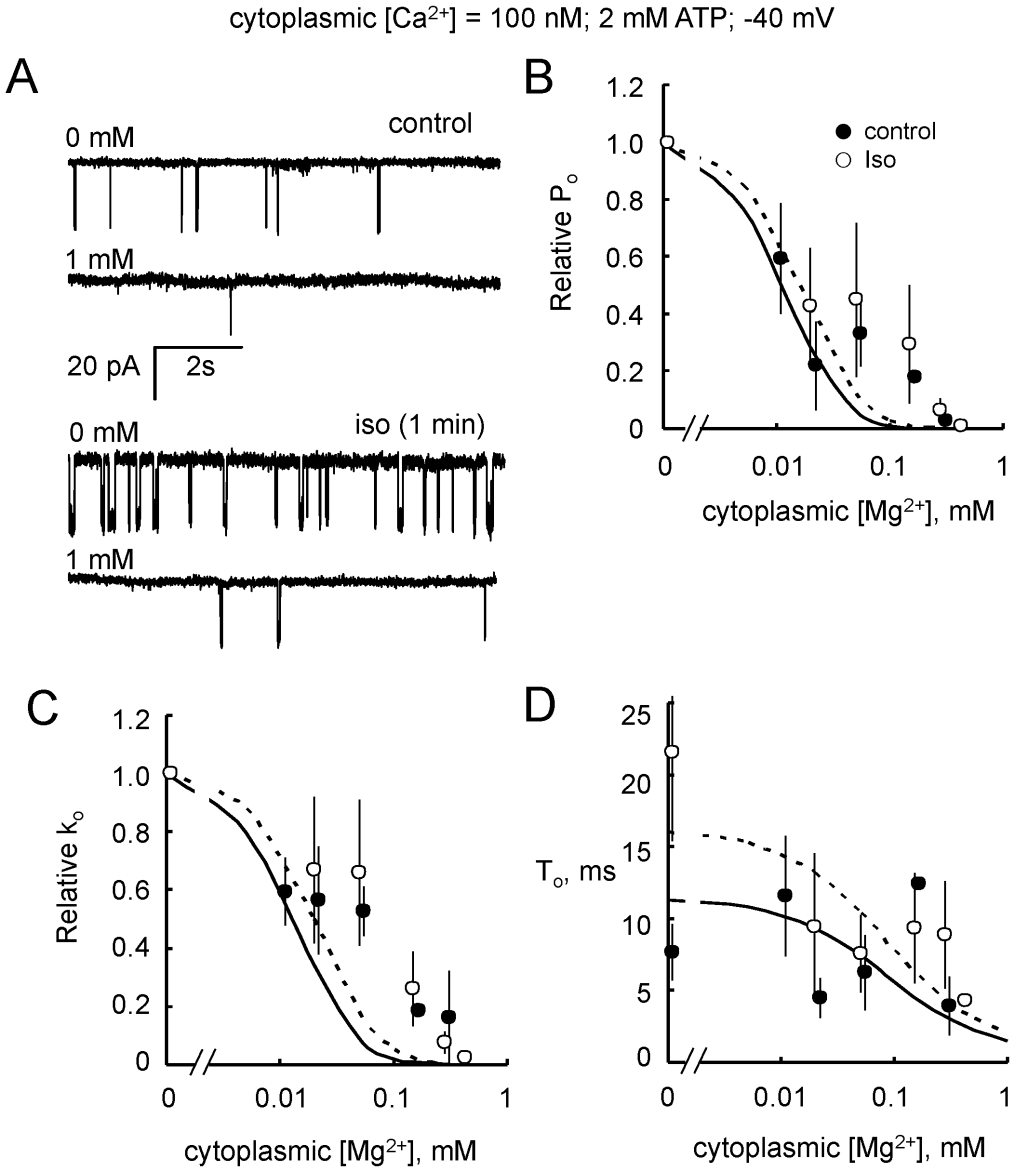
Inhibition of RyR2 by cytoplasmic Mg^2+^. (**A**) Single channel recordings of RyR2 from control (Traces 1 & 2) and isoproterenol stimulated hearts (1 min, Traces 3 & 4) in the presence of cytoplasmic 2 mM ATP and with cytoplasmic [Mg^2+^] at the left of each trace. (**B-D**) Effects on gating kinetics of inhibition by cytoplasmic Mg^2+^ of RyR2 from control (• n = 2–10) and from 1 min isoproterenol stimulated hearts (○ n = 5–13). (**B**) Open probability, *P_o_*, and (**C**) opening rate, *k_o_*, relative to that in the absence of Mg^2+^. (**D**) [Mg^2+^]-dependencies of mean open duration, *T_o_*. Solid and dashed curves show fits of the RyR2 gating model to the control and isoproterenol data, respectively.

**Figure 6:**
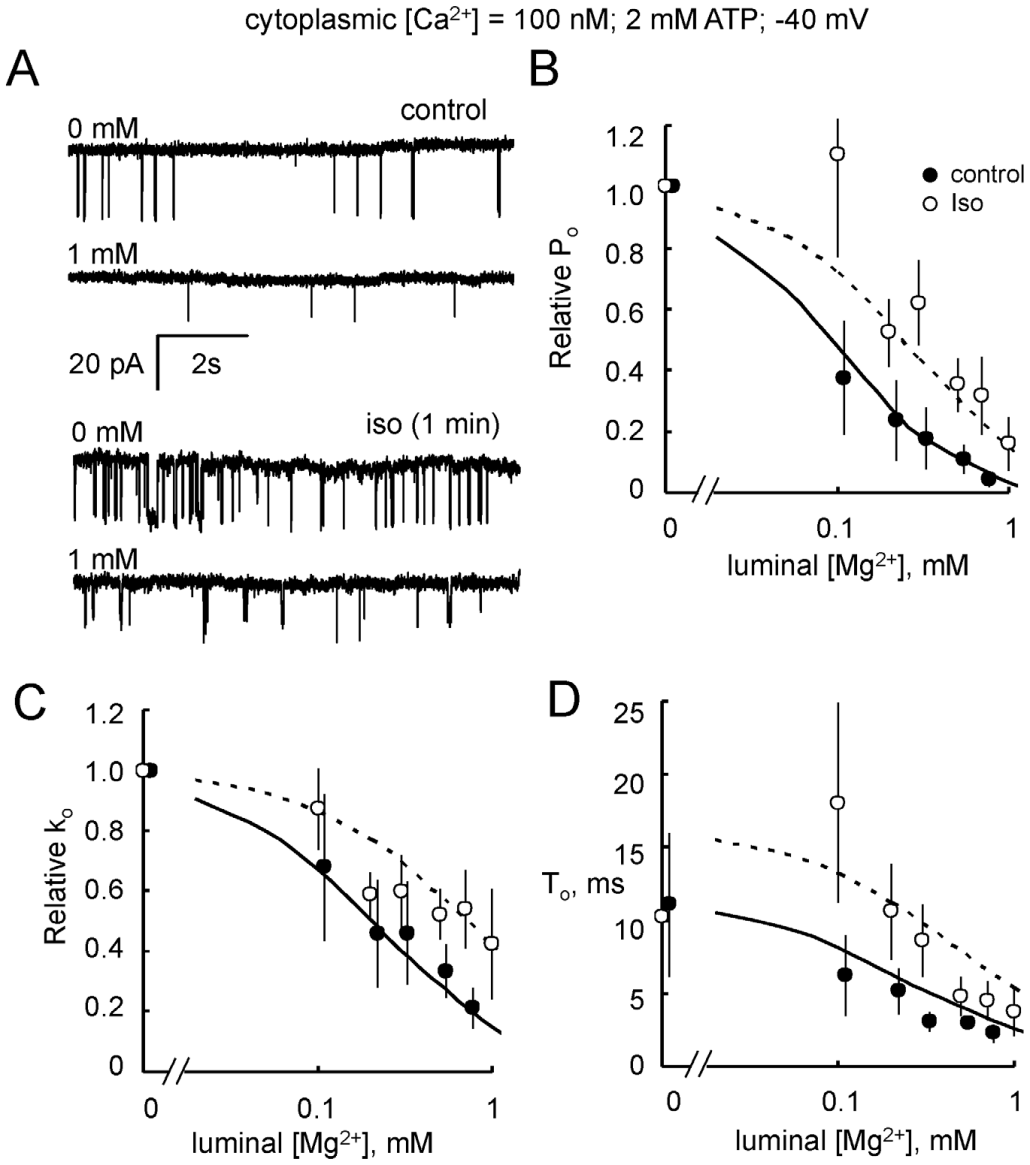
Inhibition of RyR2 by luminal Mg^2+^. (**A**) Single channel recordings of RyR2 from control (Traces 1 & 2) and isoproterenol stimulated hearts (1 min, Traces 3 & 4) in the presence of cytoplasmic 2 mM ATP and with luminal [Mg^2+^] at the left of each trace. (**B–D**) Effects on gating kinetics of inhibition by luminal Mg^2+^ of RyR2 from control (▪ n = 3–7) and from 1 min isoproterenol stimulated hearts (□ n = 3–7). (**B**) Open probability, *P_o_*, and (**C**) opening rate, *k_o_*, relative to that in the absence of Mg^2+^. (**D**) [Mg^2+^]-dependencies of mean open duration, *T_o_*. Solid and dashed curves in B show least-squares, Hill fits to the control and isoproterenol data. Solid and dashed curves show fits of the RyR2 gating model to the control and isoproterenol data, respectively.

**Figure 7:**
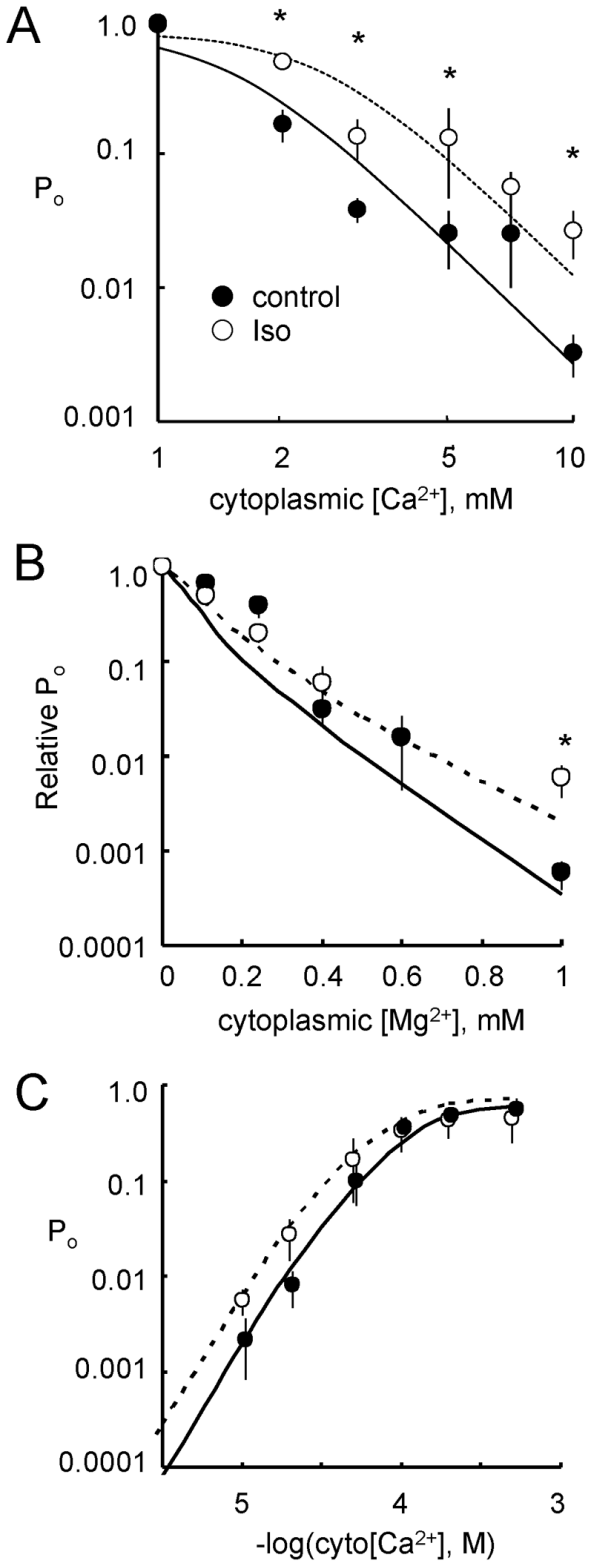
Regulation of RyR2 open probability by cytoplasmic Ca^2+^ and Mg^2+^. RyRs from control (•, n = 3–8) and 1 min isoproterenol stimulated hearts (○, n = 3–9). All recording solutions contained 2 mM ATP. (**A**) Dependence of open probability on cytoplasmic [Ca^2+^]. (**B**) Dependence of normalised open probability on cytoplasmic [Mg^2+^] in the presence of 10 µM cytoplasmic Ca^2**+**^. (**C**) Cytoplasmic Ca^2+^ activation of *P_o_* in the presence of 1 mM cytoplasmic Mg^2+^ and 1 mM luminal Ca^2+^. Asterisks indicates significant differences between • and ○ (p<0.05). Solid and dashed curves show fits of the RyR2 gating model to the control and isoproterenol data, respectively.

Cytoplasmic and luminal Mg^2+^ inhibition of RyR2 at diastolic cytoplasmic [Ca^2+^] (<1 µM) was previously shown to be due to Mg^2+^ competing with Ca^2+^ at cytoplasmic and luminal Ca^2+^ activation sites; (at higher cytoplasmic [Ca^2+^] cytoplasmic Mg^2+^ inhibits RyR2 by mimicking Ca^2+^ at the low affinity inhibition site [24] and this is dealt in the following section). Mg^2+^ inhibition via the cytoplasmic and luminal Ca^2+^ activation sites was investigated here by measuring Mg^2+^ inhibition in the presence of 100 nM cytoplasmic Ca^2+^ (Figures 5 and 6). Cytoplasmic and luminal Mg^2+^ inhibition of *P_o_* were fitted with Hill equations (not shown; Hill parameters in Table 3). RyR2 gating model is compared with the data in Figures 5, 6, and 7 (parameters in Table S3 in File S1, note that model fits did not minimise least squares differences to data in Figure 5 because fits were optimised for all data in Figures 5, 6, and 7 using the same parameters). Cytoplasmic Mg^2+^ inhibition was no different (p = 0.56) in RyRs from control and stimulated hearts (Figure 5A). However, adrenergic stimulation reduced the effects of luminal Mg^2+^ inhibition of *P_o_* (Figure 6A), increasing the *K_i_* (p = 0.003) by 4.5-fold compared with control (Table 3). The decreased luminal Mg^2+^ inhibition seen after adrenergic stimulation was reflected in increased mean open durations (Figure 6D) and a decreased effect of Mg^2+^ on the opening rate (Figure 6C). For reasons outlined in the previous paragraph, the alleviation of Mg^2+^ effect on the opening rate indicates a modulation of a luminal facing site on the RyR.

**Table 3:** Parameter values for the Hill equation derived from least-squares fits to Mg^2+^ inhibition of *P_o_* shown in Figures 5 and 6.

treatment	*P_max_*	*K_i_,* µM	*H_i_*	*n*
cytoplasmic Mg^2+^ (100 nM Ca^2+^)				
Control	0.019±0.015	13±6	0.9±0.7	10
ISO (1 min)	0.10±0.04	26±11	0.8±0.5	14
luminal Mg^2+^ (0.1 mM Ca^2+^)				
Control	0.023±0.011	78±14	1.3±0.5	7
ISO (1 min)	0.032±0.022	360±100*	1.3±1.0	12

Hill equation for inhibition by Mg^2+^ is: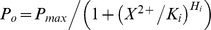.

* indicates significant difference from control value, p<0.05.

### Effect of ß-adrenergic Stimulation on Mg^2+^ Inhibition via the Cytoplasmic Ca^2+^/Mg^2+^ Inhibition Site

Cytoplasmic Ca^2+^ and Mg^2+^ can inhibit RyR2 by binding to a low affinity divalent binding site [28]. This site underlies Ca^2+^ inhibition and Mg^2+^ inhibition at mM concentrations, seen when cytoplasmic [Ca^2+^] exceeds 10 µM. Figure 7A shows Ca^2+^ inhibition of RyRs from control and stimulated hearts. Hill equations were fitted to the data, using Hill parameters in Table 2. Isoproterenol alleviated Ca^2+^ inhibition of RyRs by increasing *K_i_* from 1.5 to 2.5 mM, indicating decreased Ca^2+^ inhibition via the low affinity divalent binding site.

We also investigated effects of adrenergic stimulation on Mg^2+^ inhibition via the Ca^2+^ inhibition site by measuring Mg^2+^ inhibition of *P_o_* in the presence of 10 µM cytoplasmic Ca^2+^ (Figure 7B). We found that RyR2 from stimulated hearts showed less Mg^2+^ inhibition (at 1 mM Mg^2+^) than control, indicating decreased Mg^2+^ inhibition via the low affinity divalent binding site.

Finally, we measured the Ca^2+^ activation properties of rat RyR2s in the presence of physiological concentrations of cytoplasmic Mg^2+^ (1 mM) and in the presence of 1 mM luminal Ca^2+^ (Figure 7C). Under these conditions, the *K_a_* for cytoplasmic Ca^2+^ was 80 and 65 µM for RyRs from control and stimulated hearts, respectively. Although RyR2 from stimulated hearts showed higher mean *P_o_* than those from control hearts over the range pCa 4–5, none of the differences reached significance in these experiments.

### RyR Gating Model

Our observations are considered in the framework of a kinetic model originally developed for gating for sheep RyR2 that incorporates luminal and cytoplasmic regulation [24], [27]. We adapted the model to the rat RyR and use it to gain insight into the mechanisms of ß-adrenergic regulation of rat RyR2 by intracellular Ca^2+^ and Mg^2+^ and to predict the effect of these mechanisms on RyR activity in the cell.

**Figure 8:**
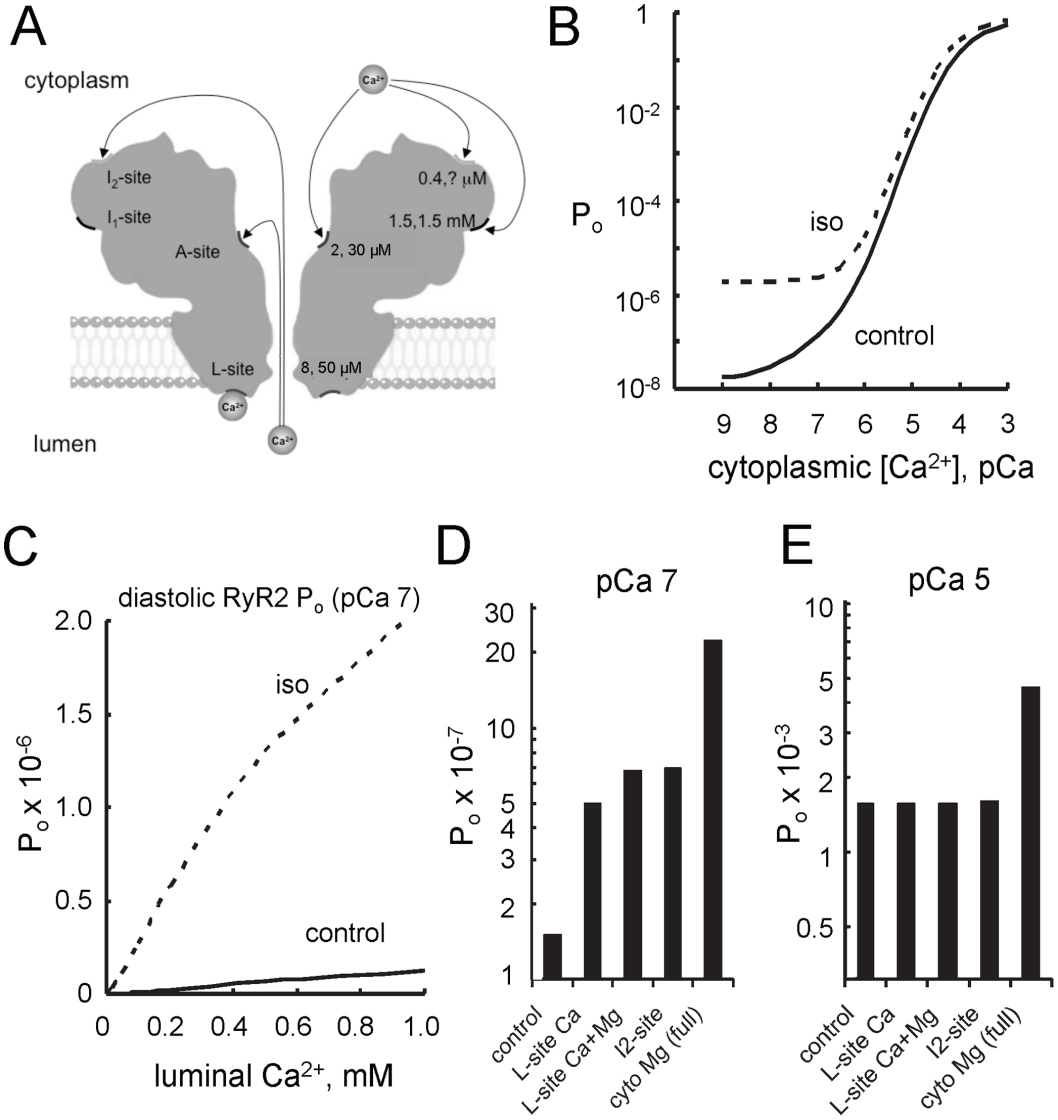
Model simulations of RyR2 open probability in ß-adrenergic stimulated and non-stimulated cells. (**A**) Ca^2+^/Mg^2+^ regulatory sites on the cardiac RyR. The hypothetical locations of four divalent cation sites known to regulate the gating activity of RyR2 are shown on a structural silhouette obtained from Samso *et al.* [41]. The names given to these sites are indicated on the left and the corresponding Ca^2+^- Mg^2+^ affinities of the sites in Rat RyR2 are shown on the right (The Mg^2+^ affinity of the *I_2_*-site is unknown). The arrows indicate the ability of Ca^2+^ on the luminal and cytoplasmic sides of the membrane to access Ca^2+^ sites on the cytoplasmic domains of the channel [42]. (**B**) Cytoplasmic [Ca^2+^] dependencies of *P_o_* with 1 mM luminal Ca^2+^ and (**C**) luminal [Ca^2+^] dependencies of *P_o_* with 100 nM cytoplasmic Ca^2+^. Simulation of RyR2 from control (solid lines) and 1 min isoproterenol stimulated hearts (dashed lines). (**D-E**) Contributions of each site to ß-adrenergic stimulation of RyR P_o_ in diastole (pCa 7; D) and systole (pCa 5; E). The categories show the cumulative effects of four adrenergic modulation mechanisms; namely: increasing Ca^2+^ activation due to the luminal L-site (L-site (Ca^2+^)), alleviating Mg^2+^ inhibition at the L-site site (L-site (Ca^2+^+Mg^2+^)), decreasing inactivation by the I_2_-site (I_2_-site) and decreasing cytoplasmic Mg^2+^ inhibition by the I_1_- and A-sites (full). The left and right categories on each bar chart show *P_o_* for control and adrenergic stimulated RyRs (control and full, respectively). The relative contributions of these sites to adrenergic stimulation are different at diastolic and systolic concentrations of cytoplasmic Ca^2+^.

The RyR2 gating model considers Ca^2+^/Mg^2+^ regulation of RyR2 in terms of four Ca^2+^ sensing sites (L-, A-, I_1_- and I_2_-sites, see Figure 8A). The experimental evidence for each of these sites is reviewed elsewhere [26], [27]. Two Ca^2+^-activation sites in the luminal (L-site) and cytoplasmic domains (A-site) of RyR2 have Ca^2+^ affinities of 8 µM and 2 µM, respectively. These sites trigger a common gating mechanism to produce synergistic activation by luminal and cytoplasmic Ca^2+^. The cytoplasmic domain also possesses two inhibitory sites with Ca^2+^ affinities of 0.4 µM (I_1_-site) and 1.5 mM (I_2_-site). Magnesium, which competes with Ca^2+^ at the L-, A- and I_2_-sites, inhibits RyRs and shapes the Ca^2+^-dependent activation of RyRs [24]. Luminal Ca^2+^ and Mg^2+^ can pass through open channels and act at cytoplasmic facing sites.

RyR opening rates and mean open durations (Figures 3, 4, 5, and 6) were fitted by the model (solid and dashed lines; fit parameters in Tables S2 and S3 in File S1). The model accounted for most of the key features of the data with the exception of the high RyR opening rate observed in the presence of 100 µM cytoplasmic Ca^2+^ (Figure 3C). None the less, the model did provide good predictions of *P_o_* and *T_o_* under this condition. Changes in RyR2 activity with adrenergic stimulation were reflected in changes in four parameters associated with three RyR2 sites, namely: 1) The opening rate in response to Ca^2+^ binding to the luminal Ca^2+^ activation site (L-site) increased 20-fold, accounting for the increased opening rate at low cytoplasmic [Ca^2+^] (Figure 3C) and increased luminal Ca^2+^ activation of opening rate (Figure 4C). 2) There was a 4-fold decrease in the Mg^2+^ affinity of the L-site, accounting for the decrease in luminal Mg^2+^ inhibition (Figure 6C), 3) RyR2 closing via the high affinity Ca^2+^ inactivation site (I_2_-site) was slowed 2-6-fold, accounting for the increase in mean open duration (Figure 4D). 4) The affinity of the cytoplasmic Ca^2+^/Mg^2+^ inhibition site (I_1_-site) was reduced by 40%, accounting for the reduced Ca^2+^ inhibition (Figure 7A) and Mg^2+^ inhibition in the presence of 10 µM cytoplasmic Ca^2+^ (Figure 7B). Interestingly, we found no effect of adrenergic stimulation on the cytoplasmic Ca^2+^ activation site (A-site). Model fits to the data indicated a 25% change in the Mg^2+^ affinity of the A-site which accounted for the systematic, though non-significant increase in RyR activity in Figure 7C.

We then predicted how the activity of RyRs might respond to ß-adrenergic stimulation within the cell using assumptions for the cell interior that have been reviewed and justified elsewhere [24]. Briefly, we assumed that 1) the free Mg^2+^ in cytoplasm and lumen is 1 mM, 2) Ca^2+^ and Mg^2+^ fluxes through RyR2 are slightly larger in the presence of intracellular [K^+^] than with 250 mM Cs^+^ and that 3) intracellular Ca^2+^ buffering is substantially weaker and slower in the cell than in our bilayer solutions. Assumptions 2 and 3 affect the feed-through parameters, *X_A_* and *X_I_* in the model (see Table S3 in File S1).

The model predicts that under systolic conditions where cytoplasmic [Ca^2+^] exceed 1 µM, ß-adrenergic stimulation causes a 3-fold increase in RyR2 *P_o_* (Figure 8B). This is similar to a 2-fold stimulation of *P_o_* measured at systolic levels of Ca^2+^ and Mg^2+^ (Figure 7C). Under diastolic conditions (cytoplasmic [Ca^2+^] ∼100 nM), the model predicts a RyR2 open probability in the range 10^−7^ to 10^−6^ which is far too small to be measured by single channel recording. Under these conditions, ß-adrenergic stimulation increases RyR2 *P_o_* by 20-fold, which was due to an increase in the ability of luminal Ca^2+^ to activate the RyR (Figure 8C).

We explored how the modulation of the L-, I_1_- and I_2_-sites during ß-adrenergic stimulation contribute to increasing RyR2 *P_o_* under both diastolic (Figure 8D) and systolic conditions (Figure 8E). In diastole, the model predicted that the increased action of Ca^2+^ binding to the L-site contributes a 3-fold increase in RyR2 activity during ß-adrenergic stimulation (Figure 8D, L-site (Ca^2+^)), decreased Mg^2+^ inhibition at the L-site contributed another 50% increase (Figure 8D, L-site (Ca^2+^+Mg^2+^)) and reduced cytoplasmic Mg^2+^ inhibition contributed another 3-fold increase in activity (Figure 8D, full; I_1_-site and A-site contributed ∼1.7-fold each). The I_2_- and A-sites made no significant contribution to ß-adrenergic stimulation. In systole, the only significant contribution to ß-adrenergic stimulation came from a decrease in cytoplasmic Mg^2+^ inhibition (Figure 8E).

## Discussion

### Effect of ß-adrenergic Stimulation on RyR2 Regulation by Ca^2+^ and Mg^2+^

Here, we report the first characterization of steady-state changes in RyR2 regulation by cytoplasmic and luminal Ca^2+^ and Mg^2+^ in response to acute ß-adrenergic stimulation of the heart by isoproterenol. We find that ß-adrenergic stimulation is associated with a marked change in RyR2 regulation by intracellular Ca^2+^ and Mg^2+^ and an increase in the level of phosphorylation of S2808 and S2814 on the RyR. ß-adrenergic-induced changes in RyR function were reversed when RyR2 was dephosphorylated by incubation with PP1, indicating that ß-adrenergic-induced changes were mediated by phosphorylation.

Our findings are consistent with, and substantially extend previous observations that RyR2 from ß-adrenergic stimulated mouse hearts that had a higher opening rate than unstimulated hearts [2], [6], [29] at diastolic cytoplasmic Ca^2+^ (150 nM) and supra-physiological luminal Ca^2+^ (50 mM). However, the degree of RyR activation seems to depend on the presence of ATP and Mg^2+^. In the absence of ATP and Mg^2+^, isoproterenol stimulation caused a 15-fold increase in RyR2 activity and less than a 2-fold increase in the presence of ATP [6]. Another study [29] reported that exercise caused a 5-fold increase in RyR activity in the presence of Mg^2+^. Here, we present the first investigation of how ß-adrenergic stimulation alters the various Ca^2+^ and Mg^2+^ regulation mechanisms in RyR2 and their relative roles in physiological Ca^2+^ release from the SR. We identify four actions of ß-adrenergic stimulation on RyR2 gating which we associate with three mechanisms: #1) a 3- to 5-fold increase in RyR2 activation by luminal Ca^2+^ (Figure 4) and decreased RyR2 inhibition by luminal Mg^2+^ (Figure 6); both actions being attributable to changes in the luminal Ca^2+^ binding site (L-site) [24]. #2) diminished Mg^2+^ inhibition at mM concentrations (Figure 7) attributable to decreased affinity of the I_1_-site and possibly the A-site, and #3) increased RyR2 mean open durations (Figure 4D), attributable to a decreased rate of cytoplasmic Ca^2+^ inactivation (I_2_-site). The cytoplasmic Ca^2+^ activation, associated with the cytoplasmic Ca^2+^ activation site (A-site; [28]) was not altered by ß-adrenergic stimulation. This novel data show that changes in luminal Ca^2+^ activation make the dominant contribution to increases in *P_o_* observed at low cytoplasmic [Ca^2+^] whereas at higher [Ca^2+^], the dominant contribution comes from alleviation of cytoplasmic Mg^2+^ inhibition.

By fitting the data obtained here from rat RyR2 with a model previously used to account for Ca^2+^ and Mg^2+^ regulation of sheep RyR2 [24] and extrapolating this model to physiological ionic and Ca^2+^ buffering conditions, we predict that different combinations of these mechanisms underlie stimulation of RyR2 activity in diastole and systole. In diastole, ß-adrenergic stimulation causes a 20-fold increase in RyR *P_o_*, mainly due to mechanisms #1 and #2 whereas in systole, it causes a smaller, 3-fold increase in *P_o_*, mediated by mechanism #2.

### Phosphorylation of RyRs during ß-adrenergic Stimulation

Western Blot analysis shows that ß-adrenergic stimulation of intact hearts induces a 4-fold increase in S2814 phosphorylation, which is at the high end of the range reported by previous studies of perfused hearts [30], [31]. In addition, S2814 phosphorylation occurs within 60 s of ß-adrenergic stimulation suggesting a role in the acute changes in cardiac function induced by isoproterenol. Several studies show that S2814 phosphorylation occurs as a result of the raised cytoplasmic [Ca^2+^] resulting from increased heart rate during adrenergic stimulation which activates CaMKII [2], [32].

Western blots with pS2808 antibody indicated that S2808 was substantially phosphorylated before ß-adrenergic stimulation and that ß-adrenergic stimulation increased phosphorylation at this site (Figure 1). We used the DepS2808 antibody that detected dephosphorylated S2808 as a cross check for the pS2808 experiments. The experiments using DepS2808 confirmed the high basal levels of S2808 phosphorylation but not the effects of ß-adrenergic stimulation due to the low levels of antibody staining in these experiments. Our pS2808 data confirm antibody measurements of basal and ß-adrenergic-induced phosphorylation levels of S2808 in rat perfused hearts [30], quiescent cardiomyocytes [33], [34] and other species [19], [21], [31], [34], [35]. However, studies using ^32^P incorporation assays report larger relative increases in S2808 phosphorylation that imply lower basal phosphorylation levels than we report. Exercise in mice [29] and isoproterenol administered to rats [6] induced 3-fold increases in PKA phosphorylation in RyR2.

We did not examine phosphorylation at S2030 because we could not detect pS2030 antibody binding to RyR2. Previous characterization of several antibodies by other workers also failed to reliably measure S2030 phosphorylation [33]. However, ß-adrenergic-induced changes in phosphorylation at S2030 were detected by others in mouse and rat using antibodies not available to us [31], [34]. Potential changes in phosphorylation at S2030 would not affect our main conclusions regarding how RyR2 gating changes in response to ß-adrenergic stimulation.

### Synergistic Activation of RyR by Phosphorylation at Multiple Sites

A picture is emerging where phosphorylation at any of the three known phosphorylation sites on the RyR2 increases its activation by luminal Ca^2+^. The ß-adrenergic-induced increase in luminal Ca^2+^ activation, that correlated in this study with increased phosphorylation of S2814 and S2808, is similar to that reported by constitutive phosphorylation of S2030 in recombinant S2030D mice [9], [14] and PKA-induced phosphorylation of S2808 [36]. ß-adrenergic stimulation of the heart is substantially reduced in S2808A (dephosphorylated) transgenic mice [36], [37]. These apparently conflicting findings could be reconciled if multiple phosphorylation sites have a synergistic effect on RyR2 activity and that adrenergic stimulation requires phosphorylation at both S2814 and S2808. This would not be surprising given the close proximity of S2808 and S2814 in the RyR2 sequence.

### RyR Activity during ß-adrenergic Stimulation Compared with Manipulation of Phosphorylation by Exogenous Enzymes

Regulation of RyR2 by phosphorylation and luminal Ca^2+^ and Mg^2+^ is controversial. Our finding that the effect of ß-adrenergic stimulation strongly correlates with CaMKII phosphorylation at S2814 and that its effect on RyR2 activity depends on the ionic conditions used in bilayer experiments appears to reconcile some conflicting reports using exogenous CamKII to phosphorylate RyRs. For example, the ß-adrenergic-induced increase in RyR2 luminal Ca^2+^ activation in the presence of 100 nM cytoplasmic Ca^2+^ was found in other studies of S2814 phosphorylation by CaMKII [38] and the phospho-mimetic S2814D mutation [2]. The contradictory findings that CaMKII had either no effect on RyR2 activity [38], or caused a decrease in activity [39] were obtained using cytoplasmic Ca^2+^ in excess of 1 µM. Under such conditions we also find that ß-adrenergic stimulation also had no effect on RyR2 activity. We could not detect an effect of adrenergic stimulation on RyR2 inhibition by cytoplasmic Mg^2+^ in the presence of 100 nM cytoplasmic Ca^2+^; a phenomenon associated with the A-site [28]. However, ß-adrenergic stimulation did reduce Mg^2+^ inhibition at high cytoplasmic Ca^2+^ (associated with the I_1_-site); consistent with the finding that exogenous CaMKII removes Mg^2+^ inhibition with 5–10 µM Ca^2+^ [8], [25]. Hain *et al.* [25] found that activation of endogenous CaMKII caused inhibition of RyRs that were previously dephosphorylated by PP1. In our study, ß-adrenergic activation of RyR2 associated with CamKII activity could be because RyR2 in our experiments were not dephosphorylated prior to stimulation.

### Technical Limitations

The aim of this study was to identify the effects of acute ß-adrenergic stimulation on RyR2 function that occur in a timeframe that similar to the rate of onset of ß-adrenergic stimulation in the heart. To achieve this it was necessary adopt the *in vitro* Langendorff perfusion method so that hearts could be snap frozen within one minute of administering isoproterenol. (Flash freezing methods have been found to accurately capture the phosphorylation state of RyR2 [6]). Sustained perfusion (15 min) of isolated hearts appears to reduce phosphorylation levels of RyR2 compared to hearts *in situ* (*c.f.* Fig 1A,C in [6]). Therefore, control hearts in our study were perfused for just long enough for heart rate to reach a steady level (5 min). This approach would minimise run-down in RyR2 phosphorylation during perfusion but at the cost of ensuring that hearts have fully recovered from explantation.

We find that the binding of antibodies for S2808 and S2814 to RyRs that were incubated with PKA and CamKII phosphorylation indicated that S2808 was mainly phosphorylated by PKA and S2814 by CamKII. In addition, these kinases appeared to phosphorylate the alternate sites to 20–50%, confirming previous antibody experiments [19] indicating that PKA and CamKII act on both sites. However, given that ^32^P incorporation experiments find that PKA and CamKII are each highly specific for S2808 and S2814, respectively [40], we can’t rule out the possibility that our antibodies are cross-reacting with both phosphorylated serines.

In conclusion, ß-adrenergic stimulation induces changes in at least three Ca^2+^ and Mg^2+^ regulation mechanisms in RyR2. In bilayer experiments, these changes produce changes in channel activity that depended on the experimental conditions, which can account for conflicting reports of phosphorylation-induced changes in RyR2 function. In the cell, our RyR2 gating model predicts that ß-adrenergic stimulation of RyR2 is 20-fold in diastole but only 3-fold in systole; reflecting the different mechanisms underlying stimulation in each case.

## Supporting Information

File S1 **Supplementary material detailing the methods and the model for Ca^2+^/Mg^2+^ dependent gating of RyR2.** The file gives additional information for methods of 1) perfusion of isolated rat hearts, 2) data acquisition and analysis, 3) SDS PAGE and Western Blotting, 4) in-vitro Exogenous PP1, PKA and endogenous CaMKII activity assays and 5) a summary of heart rates and phosphorylation levels determined by Western Blots for each heart used in this study (Table S1). Additional details of the model are included in Tables S2 and S3.
(DOCX)

File S2 **Figures of supporting information on the analysis of phosphorylation of RyR2 in Western Blots.** These figures demonstrate 1) the feasibility of reprobing Western Blots, 2) the time course of PP1 incubation of SR vesicles, 3) the time course of binding by antibodies to phospho-S2808 and phospho-S2814 in response to PKA and CamKII incubation, 4) that endogenous phosphatases do not alter S2808 phosphorylation in bilayer experiments and 5) that ATP does not alter S2814 phosphorylation in bilayer experiments.
(PDF)
