# Supplementary material for: ß-Adrenergic Stimulation Increases RyR2 Activity via Intracellular Ca2+ and Mg2+ Regulation
Source: PLoS One. 2013 Mar 22;8(3):e58334. doi: 10.1371/journal.pone.0058334 (PMC3606165; doi:10.1371/journal.pone.0058334)
Supplement: File S1 — Supplementary material detailing the methods and the model for Ca2+/Mg2+ dependent gating of RyR2. The file gives additional information for methods of 1) perfusion of isolated rat hearts, 2) data acquisition and analysis, 3) SDS PAGE and Western Blotting, 4) in-vitro Exogenous PP1, PKA and endogenous CaMKII activity assays and 5) a summary of heart rates and phosphorylation levels determined by Western Blots for each heart used in this study (Table S1). Additional details of the model are included in Tables S2 and S3. (DOCX) [file pone.0058334.s001.docx]

**METHODS**

***Perfusion of Isolated Rat Hearts***

Healthy male adult rats (Sprague-Dawley, 12-14 weeks) were heparinized (2000U, Heparin Injection BP, Weddel Pharmaceuticals Ltd.) and 15 minutes later anesthetized by Isoflurane (Laser, Australia) as approved by the University of Newcastle Animal Care and Ethics Committee. The hearts were rapidly removed and immediately perfused via the Langendorff method with ice-cold Krebs Henseleit buffer (in mM: 120 NaCl, 25 NaHCO_3_, 10 Glucose, 5 KCl, 2 MgCl_2_, 1 NaH_2_PO_4_, 2.5 CaCl_2_) for 2 min until the blood was washed out. Hearts were then perfused with warmed (37°C) and oxygenated (5% O_2_-5% CO_2_) Krebs Heseleit buffer for 5 min after which they were either perfused with buffer containing 1 μM isoproterenol (Sigma) or no isoproterenol. During perfusion, heart performance was monitored using a physiological pressure transducer (Gould-Statham Instruments) and recorded through PowerLab Chart version 5.2 (AD Instruments). Following perfusion, the whole hearts were quickly frozen in liquid nitrogen in order to preserve their phosphorylation state and then stored at -80°C.

***Data Acquisition and Analysis***

Electrical potentials were expressed with respect to the luminal side as ground. During experiments the channel currents were recorded using a 50 kHz sampling rate and 5 kHz low pass filtering. Prior to analysis the current signal was digitally filtered at 1 kHz with a low-pass Gaussian digital filter. Channel 2 software (P.W. Gage and M. Smith, Australian National University, Canberra) was used to detect channel events and measure open probability (*P_o_*), mean open duration (*T_o_*) and mean closed duration (*T_c_*). Events were detected using 50% threshold crossing method. Opening rate (*k_o_*) was calculated as: *k_o_*=1*/T_c_*. For experiments in which bilayers contained several RyRs, *P_o_* was calculated from the time-averaged current divided by the unitary current and the number of channels (*n*). Both methods of calculating *P_o_* gave similar results. The mean channel open and closed durations (*T_o_* and *T_c_*) could also be calculated from mean open and closed durations in multi-channel recordings (*T_o_*(*n*) and *T_c_*(*n*)) provided that multiple openings were rare: *T_o_* = *T_o_*(*n*) and *T_c_*=*T_c_*(*n*) x *n*. The number of channels in each experiment was determined during periods of strong activation, which were usually achieved by turning off the local perfusion and exposing the RyRs to the *cis* bath. Simulations of *T_o_* and *T_c_* were generated from theoretical models using the ‘Q-matrix’ method of Colquhoun and Hawkes [[1](#_ENREF_1),[2](#_ENREF_2)]. We accounted for the effects of missed events due to filtering using the ‘effective rate constant’ method of Blatz and Magleby [[3](#_ENREF_3)].

***SDS PAGE and Western Blotting***

SR proteins (7.5 μg) were loaded onto NuPAGE Novex Tris-Acetate 3-8% gradient gels and transferred to nitrocellulose membranes using an iBlot® transfer device (Invitrogen). Gels were pre-soaked in equilibration buffer for 20 min before transferring in accordance with the manufacturers’ recommendations. Following blocking for 1 h with PBS containing 3% Bovine Serum Albumin (BSA; Sigma), membranes were incubated with either anti-RyR2phospho-S2808 (pS2808; 1:4000), anti-RyR2dephospho-S2808 (DepS2808; 1:800), anti-RyR2phospho-S2814 (pS2814; 1:800) or anti-RyR2 C3-33 (1:1000) monoclonal antibodies in PBS containing 0.1% Tween-20, at 4°C overnight. Anti-RyR2 (C3-33) antibody was purchased from Abcam, and others were from Badrilla UK. After washing, membranes were incubated with anti-rabbit IgG HRP-conjugated secondary antibodies (1:5000; Santa Cruz) or mouse immunoglobulins (1:5000; BioRad) for 1 h, and then incubated with enhanced chemiluminescence reagent (ECL Plus Kit; GE Healthcare) and visualized using a LAS-4000 camera system (Fuji Life Sciences). Between all steps membranes underwent 3 x 5 min rinses with PBS containing 0.1% Tween-20. Densitometric analysis was undertaken using Multi Gauge image acquisition and analysis software (version 3). To establish loading controls, each membrane probed with phospho-antibodies was re-probed with anti-RyR2 (C3-33 from Abcam) after quenching of HRP activity with sodium azide (3% w/v for 3 h at room temperature (Figure 1).

*In-vitro Exogenous PP1, PKA and Endogenous CaMKII Activity Assays*

Time-dependent activity of exogenous PP1, PKA and endogenous CaMKII on RyR2 from control hearts was measured for maximal dephosphorylation and phosphorylation of control RyR2 as controls in quantitative western blot. In-vitro PP1 activity on RyRs from control heart was carried out using a PP1 reaction kit (New England BioLabs) containing MnCl_2_, NEBuffer® and 2.5 unit/μl PP1 at 30°C. The degree of S2808 phosphorylation was measured at 5 min PP1-exposure intervals for 30 min (Figure 2). To measure in-vitro PKA activity on RyRs from control heart, RyRs were first maximally dephosphorylated by PP1 (15 min incubation as above) and then incubated in PKA buffer containing (mM): 50 Tris-HCl (pH 7.4), 10 MgCl_2_, 2 ATP, 1 cAMP, 10 NaF and 0.25 g/ml PKA (Sigma Aldrich) at 30°C. RyRs incubated with PKA for 25 min were again incubated in a CaMKII activating buffer containing (mM): 50 Tris-HCl (pH 7.4), 10 MgCl_2_, 2 ATP, 25 NaF, 2.5 CaCl_2_ and 62.5 μM Calmodulin at 30°C for 20 min, and this is used as a measure of maximal phosphorylation by PKA and CaCaM. The CaMKII activation buffer contained no exogenous CaMKII because endogenous CaMKII is known to be associated with and phosphorylate the RyR2 in vitro [[4-6](#_ENREF_4)]. During these incubations, S2808 and S2814 phosphorylation was measured at 5 min intervals by sequentially probing (and stripping, see above) membranes with anti-pS2808, anti-pS2814 and C3-33 antibodies (loading control, Figure 3A). A measure of the specificity of antibody binding for phosphorylated S2808 and S2814, blot densities were normalised to that for maximal phosphorylation by PKA and CaCaM (Figure 3B). Conversely, to measure in-vitro CaMKII activity on RyRs from control heart, RyRs were first maximally dephosphorylated by PP1 and then incubated in a CaMKII activating buffer and then PKA (Figure 4). In each case, the reactions were stopped by adding LDS sample buffer (Invitrogen).

To determine whether endogenous phosphatases and CaMKII could alter RyR2 phosphorylation in bilayer experiments, SR vesicles were incubated in bilayer cis solution (without and with ATP, Figures 5 and 6, respectively) for up to 30 min Blots were first probed with either pS2808 or pS2814 antibody as appropriate. Following membrane strip in PBS with 2% SDS and 100 mM β-mercaptoethanol and reblock of the membrane, blots were probed with anti-RyR2 antibody, to determine the amount of RyR2 transferred.

**TABLE S1**

| **Heart ID** | **Basal HR (BPM)** | **HR at 1 min (BPM % increase)** | **DepS2808 density (n)** | **pS2808 density (n)** | **pS2814 density (n)** |
| --- | --- | --- | --- | --- | --- |
| *control* |  |  |  |  |  |
| 1 | 270 | - | 0.2±0.04(9) | 0.94±0.08 (4) | 0.1±0.03 (5) |
| 2 | 300 | - | 0.1±0.03 (5) | 0.8±0.1 (10) | 0.17±0.04 (3) |
| 3 | 139 | - | 0.24±0.04 (7) | 0.6±0.15 (6) | 0.22±0.07 (8) |
| 4 | 179 | - | 0.24±0.02 (12) | 0.58±0.08 (15) | 0.28±0.06 (10) |
| 5 | 331 | - | - | - | - |
| 6 | 213 | - | - | - | - |
| 7 | 248 |  |  | 0.81 (1) |  |
| 8 | 245 |  |  | 0.69±0.01(2) |  |
| *ISO 1 min* |  |  |  |  |  |
| 9 | 276 | 384(39) | 0.14±0.03 (6) | 0.83±0.08 (6) | 0.49±0.1 (5) |
| 10 | 204 | 400(96) | 0.16±0.04 (6) | 1.09±0.14 (6) | 0.72±0.04 (4) |
| 11 | 167 | 318(90) | 0.27±0.06 (6) | 0.68±0.09 (5) | 0.53±0.04 (4) |
| 12 | 209 | 408(95) | - | - | - |
| 13 | 160 | 276(73) | - | - | - |
| 14 | 180 | 300(67) | - | - | - |
| 15 | 223 | 345(55) |  |  |  |
| 16 | 222 | 309(39) |  |  |  |
| 17 | 189 | 353(87) |  |  |  |
| 18 | 254 | 355(40) |  |  |  |

**Table S1.** Summary of heart rates (HR) and phosphorylation levels determined by Western Blots for each heart used in this study. Numbers in parentheses indicate the number of Western blots carried out on samples from each heart.

**THE RYR2 Ca^2+^/Mg^2+^ DEPENDENT GATING MODEL**

TABLE S2A TABLE S2B

| Subunit | Opening rate | | Closing rate | |
| --- | --- | --- | --- | --- |
| stoichiometry | *k_o_*, s^-1^ | | *k_c_*, s^-1^ | |
|  | control | ISO | control | ISO |
| ○○  ○○ | 0 | 0 | 1000 | 1000 |
| ●● ●●  ●● ●○ | 0.1 | 1.3-2.5 * | 1000 | 1000 |
| ●● ●●  ○● ●○  ●● ●●  ●● ●● | 4 | 4 | 1000 | 1000 |
| ●● ●●  ○● ●● | 80 | 80 | 1000 | 1000 |
| ●●  ●● | 10000 | 10000 | 1 | 1 |

|  |  | A-site | | |
| --- | --- | --- | --- | --- |
|  |  | Ca^2+^ | 0 | Mg^2+^ |
| L-site | Ca^2+^ | ● | ● | ○ |
|  | 0 | ● | ○ | ○ |
|  | Mg^2+^ | ● | ○ | ○ |

**Table S2**. The model parameters for the rates of opening and closing of the RyR in response to binding of Ca^2+^ and Mg^2+^ to the A- and L-sites determined by fitting the model to RyR mean open durations and mean opening rates in Figures 3-6. **(A)** The RyR opening and closing rates associated with various subunit stoichiometries. Values are given for RyRs from control hearts and those stimulated by isoproterenol for 1 min. The asterisk indicates the parameter that depended on stimulation by isoproterenol. Each group of four shaded circles represents the number of subunits in each activation state (permutations of each groups are considered as equivalent). The shading of each circle indicates the binding state of A- and L-sites on each subunit as shown in **(B)**.

TABLE S3

| L-site | Control | ISO (1 min) |
| --- | --- | --- |
| *K_LCa_*  Affinity of the L-site for Ca^2+^ | 8 μM | 8 μM |
| *K_LMg_*  Affinity of the L-site for Mg^2+^ | 50 μM | 200 μM *** |
|  |  |  |
| A-site |  |  |
| *K_ACa_*  Affinity of the A-site for Ca^2+^ | 2 μM | 2 μM |
| *K_AMg_*  Affinity of the A-site for Mg^2+^ | 30 μM | 40 μM * |
|  |  |  |
| I_1_-site |  |  |
| *K_I1Ca_*  Affinity of the I_1_-site for Ca^2+^ | 1.5 mM | 2.5 mM * |
| *K_I1Mg_*  Affinity of the I_1_-site for Mg^2+^ | 1.5 mM | 2.5 mM * |
|  |  |  |
| I_2_-site |  |  |
| *K_I2Ca_*  Affinity of the I_2_-site for Ca^2+^ | 0.4 μM | 0.4 μM |
| *K_I2Mg_*  Affinity of the I_2_-site for Mg^2+^ | ∞ | ∞ |
| φ  opening rate associated with I_2_-site | 800 /s | 800 /s |
| θ  closing rate for I_2_-site when  3 or less A-sites bind Ca^2+^ | 2500 /s | 400-1500 /s ^a^ * |
| λ  closing rate for I_2_-site when  all 4 A-sites bind Ca^2+^ | 1.5 /s | 1.5 /s |
|  |  |  |
| *X_A_*  [Ca^2+^] at A-site due to Ca^2+^ flux through the channel | 25 μM/pA  73 μM/pA✝ | 25 μM/pA  73 μM/pA✝ |
| *Y_A_*  [Mg^2+^] at A-site due to Mg^2+^ flux through the channel | 50 μM/pA | 50 μM/pA |
| *X_I_*  [Ca^2+^] at I_2_-site due to Ca^2+^ flux through the channel | 0.35 μM/pA  29 μM/pA✝ | 0.35 μM/pA  29 μM/pA✝ |

**Table S3**. The model parameters for Ca^2+^/Mg^2+^ regulation of RyR2 gating in lipid bilayers in addition to those given in Table S2 in Apendix_S1. The asterisks indicate the parameters that depended on stimulation by isoproterenol. ✝: Parameter values under the buffering conditions in the cytoplasm. a: the value of 1500 used in fits to data in figure 3 whereas 400 was used in all other fits.

References

1. Colquhoun D, Hawkes AG (1982) On the stochastic properties of bursts of single ion channel openings and of clusters of bursts. Philos Trans R Soc Lond Biol 300: 1-59IS.

2. Colquhoun D, Hawkes AG (1981) On the stochastic properties of single ion channels. Proc R Soc Lond B 211: 205-235.

3. Blatz AL, Magleby KL (1986) Correcting single channel data for missed events. Biophys J 49: 967-980IS.

4. Bers DM (2004) Macromolecular complexes regulating cardiac ryanodine receptor function. Journal of Molecular and Cellular Cardiology 37: 417–429.

5. Currie S, Loughrey CM, Craig MA, Smith GL (2004) Calcium/calmodulin-dependent protein kinase IIdelta associates with the ryanodine receptor complex and regulates channel function in rabbit heart. Biochem J 377: 357-366.

6. Witcher DR, Kovacs RJ, Schulman H, Cefali DC, Jones LR (1991) Unique phosphorylation site on the cardiac ryanodine receptor regulates calcium channel activity. J Biol Chem 266: 11144-11152.
