# Supplementary material for: ß-Adrenergic Stimulation Increases RyR2 Activity via Intracellular Ca2+ and Mg2+ Regulation
Source: PLoS One. 2013 Mar 22;8(3):e58334. doi: 10.1371/journal.pone.0058334 (PMC3606165; doi:10.1371/journal.pone.0058334)
Supplement: File S2 — Figures of supporting information on the analysis of phosphorylation of RyR2 in Western Blots. These figures demonstrate 1) the feasibility of reprobing Western Blots, 2) the time course of PP1 incubation of SR vesicles, 3) the time course of binding by antibodies to phospho-S2808 and phospho-S2814 in response to PKA and CamKII incubation, 4) that endogenous phosphatases do not alter S2808 phosphorylation in bilayer experiments and 5) that ATP does not alter S2814 phosphorylation in bilayer experiments. (PDF) [file pone.0058334.s002.pdf]

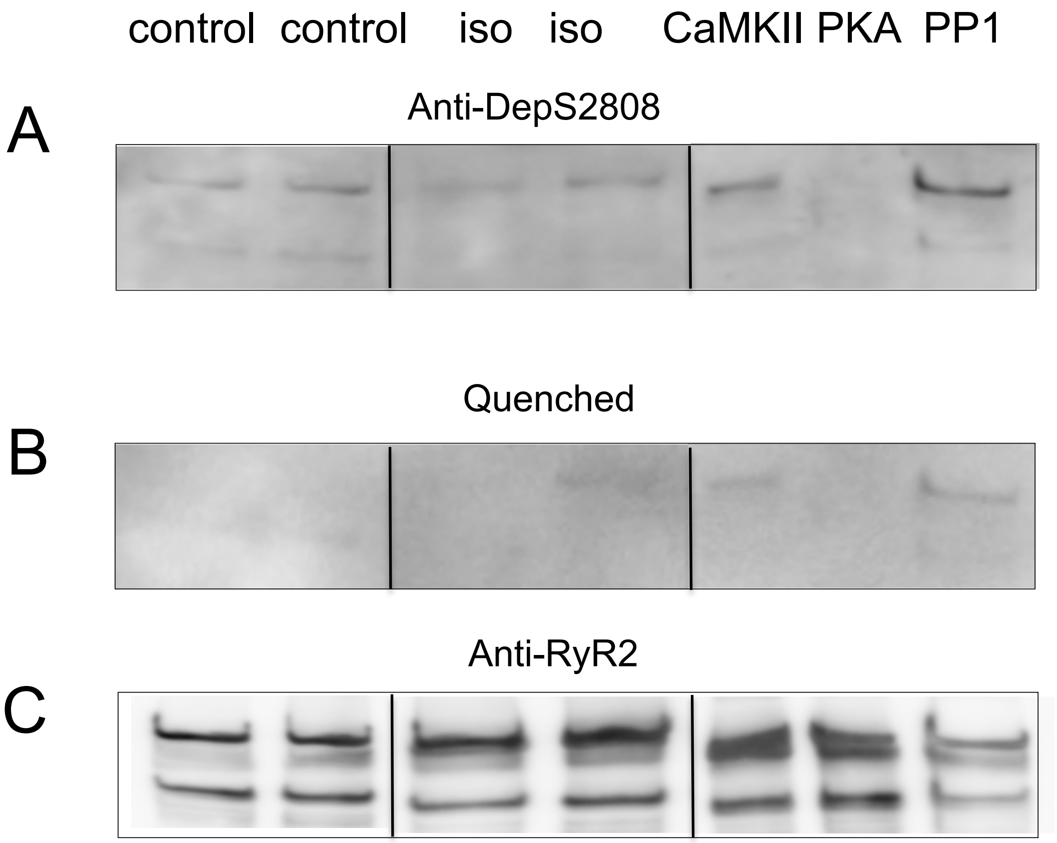

Figure S1. Analysis of dephosphorylated and total RyR2 in Western Blots. (A) Samples were first probed using DepS2808 antibodies with reactivity revealed using ECL**. (B)** After incubation with NaN_3 to inhibit Horse Radish Peroxidase activity of the secondary antibody. **(C)** Membranes were then re-probed with anti-RyR2 antibody and reactivity detected using ECL. Samples were taken from two control hearts, two isoproterenol stimulated hearts and from control samples incubated with CaMKII, PKA and PP1.

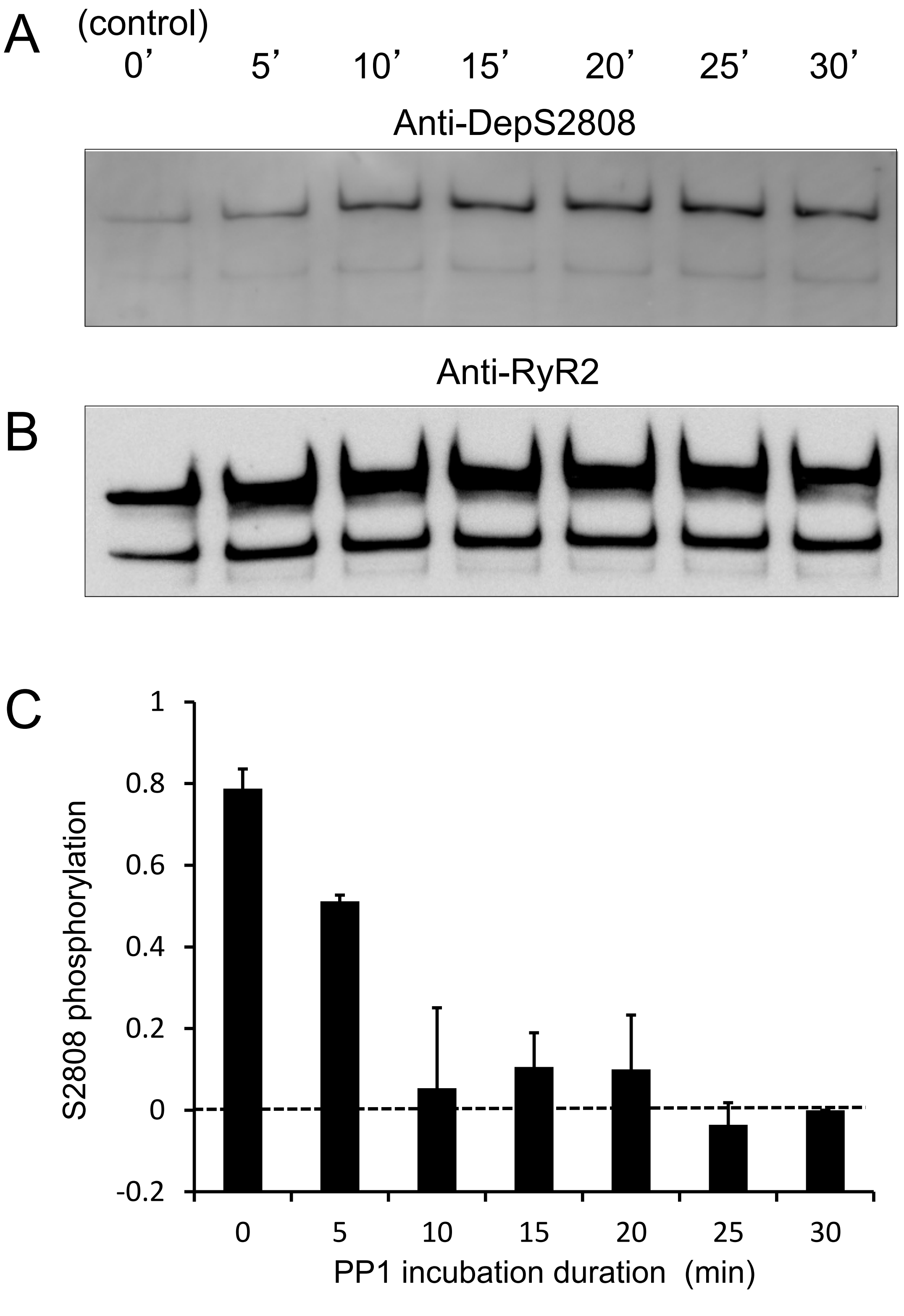

Figure S2 Time course of PP1 incubation of SR vesicles from control rat heart. (A) Western blot monitoring the phosphorylation of S2808 using the antibody for the dephosphorylated site during incubation periods given in min at 30°C**. (B)** Blots were re-probed with anti-RyR2 antibody to determine the amount of RyR2 transferred. **(C)** Summary data (n = 3) of degree of phosphorylation at S2808 relative to that seen at 30 min incubation.
Data is calculated from 1-(DepS2808/anti-RyR2).

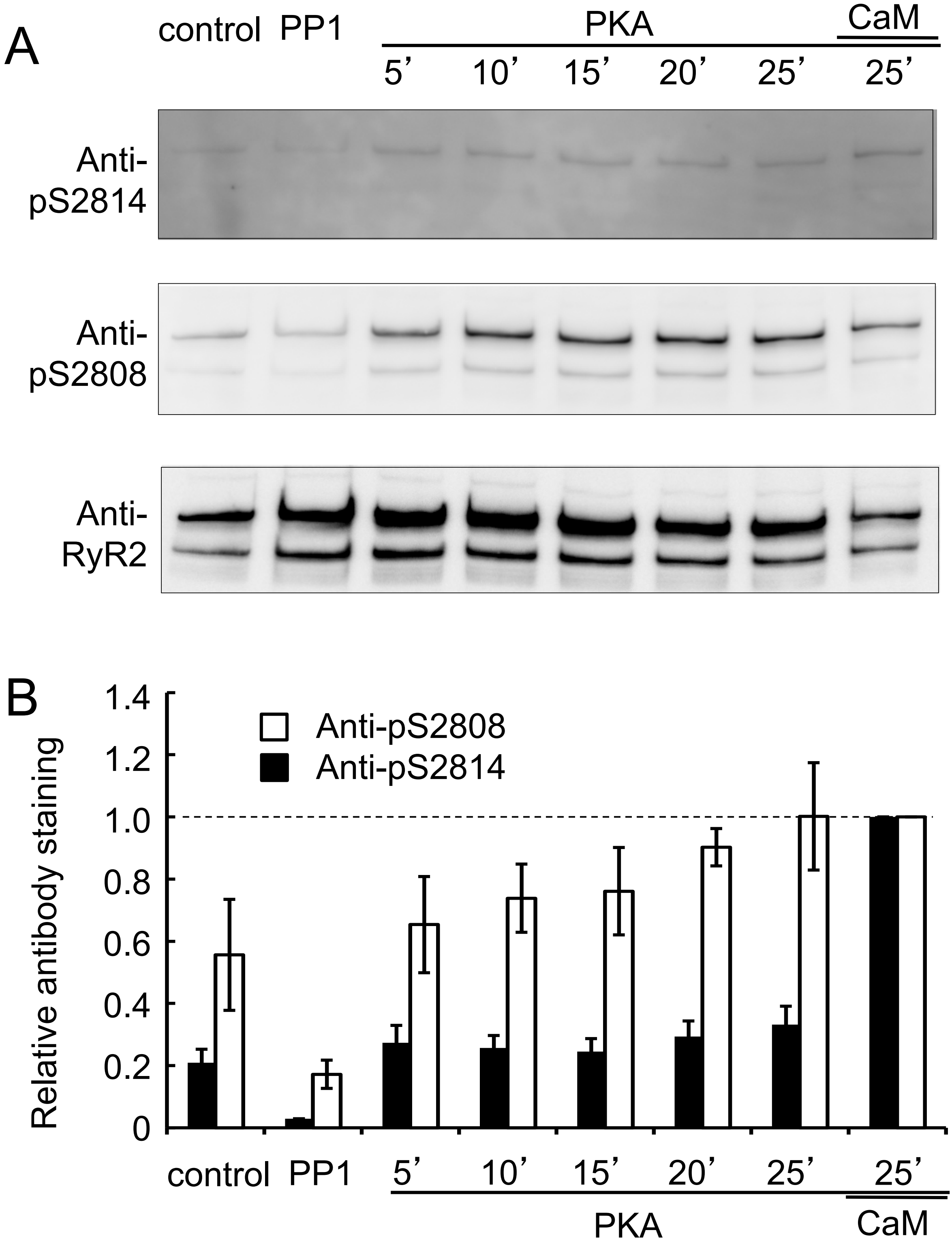

Figure S3. **Time course of binding by antibodies to phospho-S2808 and phospho-S2814 in response to PKA incubation.** RyR2 from control rat heart were dephosphorylated by 15 min incubation with PP1 followed by incubation with PKA and then PKA + CaM for 20 min. **(A)** Western blots monitoring antibody binding by pS2814, pS2808 and anti-RyR2 used as the loading control **(B)** Summary data (n=3) of antibody binding normalised to that seen by incubation with PKA and CaM.

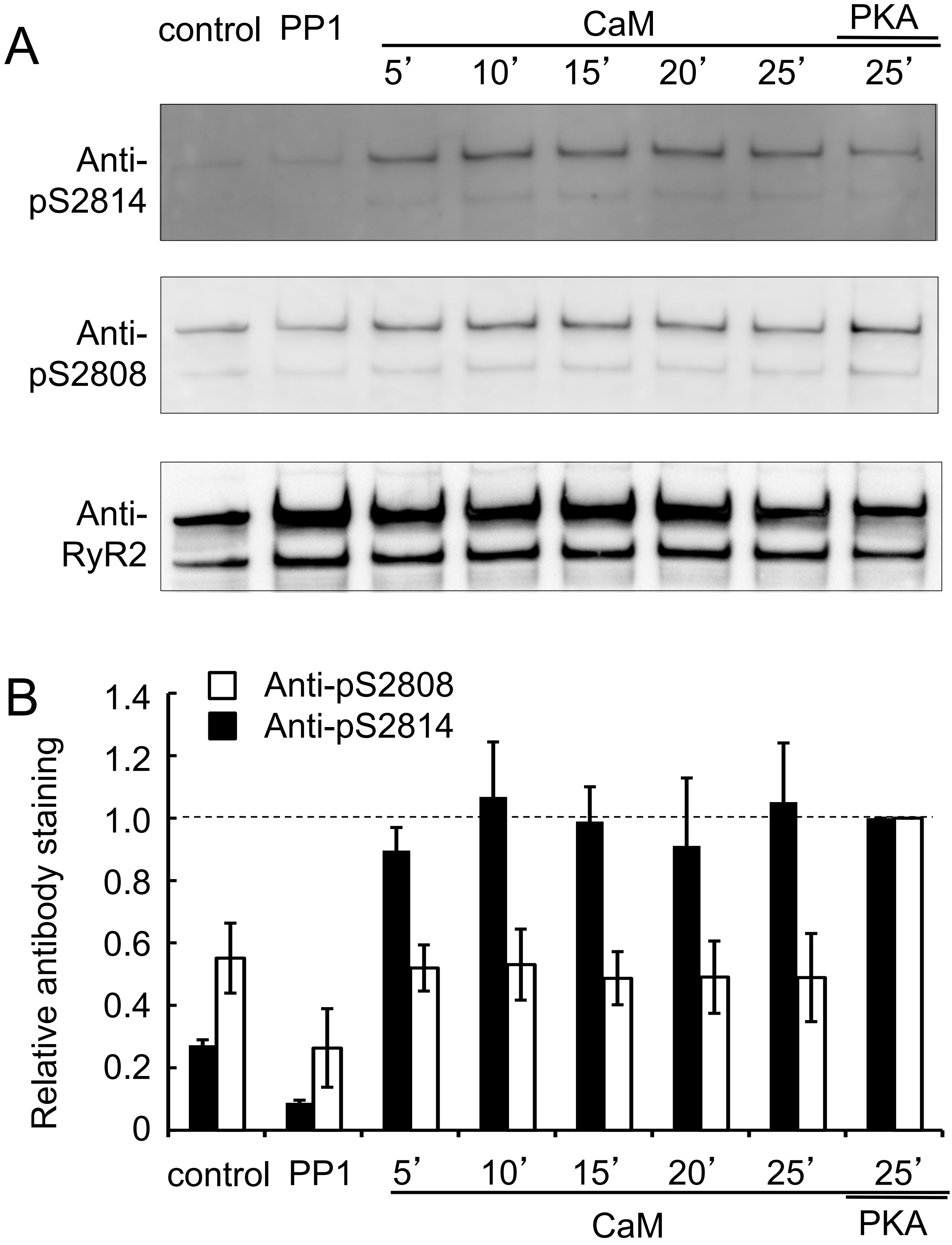

Figure S4. Time course of binding by antibodies to phospho-S2808 and phospho-S2814 in response to CaM (endogenous CamKII) incubation. RyR2 from control rat heart were dephosphorylated by 15 min incubation with PP1 followed by incubation with CaM and then CaM + PKA for 15 min. **(A)** Western blots monitoring antibody binding by pS2814, pS2808 and anti-RyR2 used as the loading control. **(B)** Summary data (n=3) of antibody binding normalised to that seen by incubation with combined PKA and CaM.

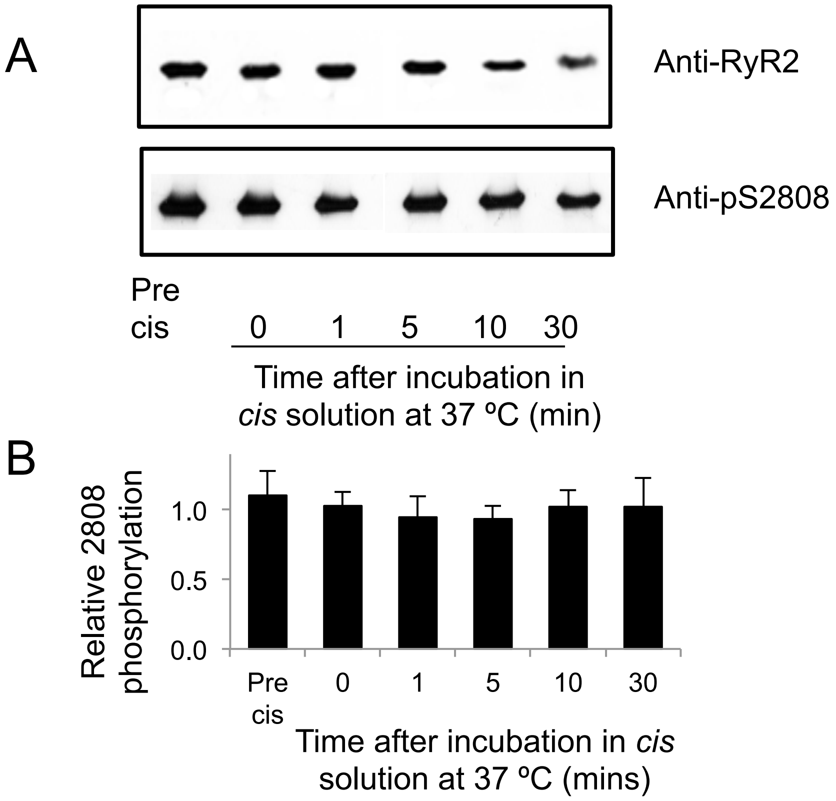

Figure S5. Endogenous phosphatases do not alter S2808 phosphorylation in bilayer experiments. (A) Western blot monitoring the phosphorylation of S2808 after incubating SR vesicles with bilayer *cis* solution containing 1 mM $CaCl_2$ for up to 30 min. Blots were first probed with pS2808 antibody and then with anti-RyR2 antibody to determine the amount of RyR2 transferred (see Methods in Appendix_S1). Separate experiments, where actin was used as a loading control (an indicator of the amount of RyR2 transferred in each lane) and RyR2 was only probed with pS2808 antibody, yielded the same results. **(B)** Average data (n = 4 separate experiments). Data has been normalized to pre-*cis* treatment levels.

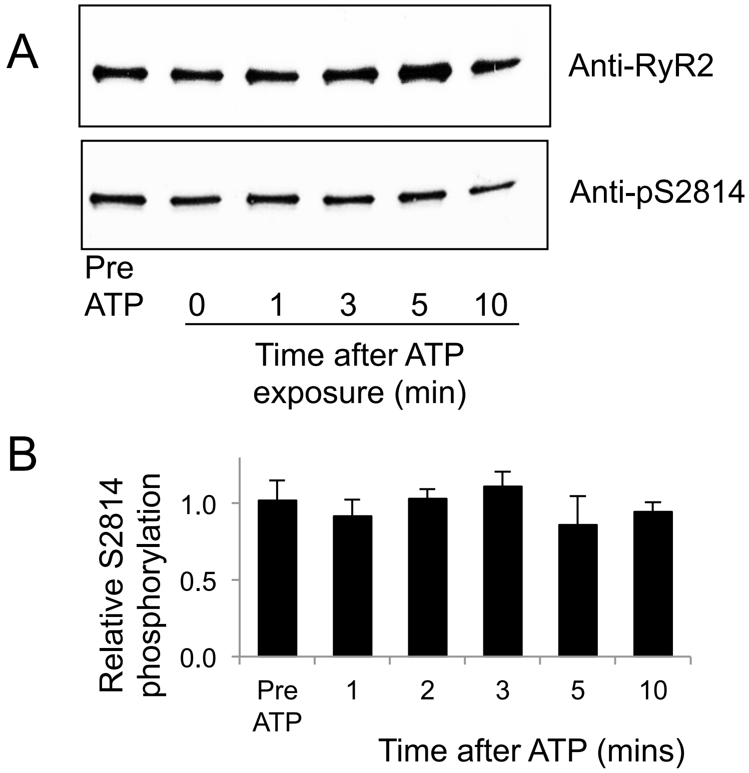

Figure S6. ATP does not alter S2814 phosphorylation in bilayer experiments. This experiment tests the possibility that S2814 on RyR2 will be phosphorylated if RyRs are exposed to ATP in the absence of exogenous calmodulin and CaMKII during single channel recording. **(A)** Western blot monitoring the phosphorylation of S2814 after incubating SR vesicles with 2 mM ATP plus 1 mM $CaCl_2$ for up to 10 min. Blots were first probed with pS2814 antibody and re-probed with anti-RyR2 antibody or actin was used as a loading control as in Figure S5 in File_S1. **(B)** Average data (n = 5 separate experiments). Data has been normalized to pre-ATP treatment levels.
